# Transcriptomic dysregulation and autistic-like behaviors in *Kmt2c* haploinsufficient mice rescued by an LSD1 inhibitor

**DOI:** 10.1038/s41380-024-02479-8

**Published:** 2024-03-26

**Authors:** Takumi Nakamura, Toru Yoshihara, Chiharu Tanegashima, Mitsutaka Kadota, Yuki Kobayashi, Kurara Honda, Mizuho Ishiwata, Junko Ueda, Tomonori Hara, Moe Nakanishi, Toru Takumi, Shigeyoshi Itohara, Shigehiro Kuraku, Masahide Asano, Takaoki Kasahara, Kazuo Nakajima, Takashi Tsuboi, Atsushi Takata, Tadafumi Kato

**Affiliations:** 1 Laboratory for Molecular Pathology of Psychiatric Disorders, RIKEN Center for Brain Science, Saitama, Japan; 2 Department of Psychiatry and Behavioral Science, Juntendo University Graduate School of Medicine, Tokyo, Japan; 3 Laboratory for Molecular Dynamics of Mental Disorders, RIKEN Center for Brain Science, Saitama, Japan; 4 Department of Life Sciences, Graduate School of Arts and Sciences, The University of Tokyo, Tokyo, Japan; 5 Institute of Laboratory Animals, Kyoto University Graduate School of Medicine, Kyoto, Japan; 6 Laboratory for Phyloinformatics, RIKEN Center for Biosystems Dynamics Research, Hyogo, Japan; 7 Laboratory for Behavioral Genetics, RIKEN Center for Brain Science, Saitama, Japan; 8 Department of Organ Anatomy, Tohoku University Graduate School of Medicine, Miyagi, Japan; 9 Laboratory for Mental Biology, RIKEN Center for Brain Science, Saitama, Japan; 10 Laboratory for Molecular Mechanism of Brain Development, RIKEN Center for Brain Science, Saitama, Japan; 11 Department of Physiology and Cell Biology, Kobe University School of Medicine, Hyogo, Japan; 12 Molecular Life History Laboratory, Department of Genomics and Evolutionary Biology, National Institute of Genetics, Shizuoka, Japan; 13 Department of Genetics, SOKENDAI (Graduate University for Advanced Studies), Shizuoka, Japan; 14 Institute of Biology and Environmental Sciences, Carl von Ossietzky University of Oldenburg, Oldenburg, Germany; 15 Department of Physiology, Teikyo University School of Medicine, Tokyo, Japan; 16 Research Institute for Diseases of Old Age, Juntendo University Graduate School of Medicine, Tokyo, Japan

**Keywords:** Autism spectrum disorders, Neuroscience, Genetics, Drug discovery, Molecular biology

## Abstract

Recent studies have consistently demonstrated that the regulation of chromatin and gene transcription plays a pivotal role in the pathogenesis of neurodevelopmental disorders. Among many genes involved in these pathways, *KMT2C*, encoding one of the six known histone H3 lysine 4 (H3K4) methyltransferases in humans and rodents, was identified as a gene whose heterozygous loss-of-function variants are causally associated with autism spectrum disorder (ASD) and the Kleefstra syndrome phenotypic spectrum. However, little is known about how *KMT2C* haploinsufficiency causes neurodevelopmental deficits and how these conditions can be treated. To address this, we developed and analyzed genetically engineered mice with a heterozygous frameshift mutation of *Kmt2c* (*Kmt2c*^+/fs^ mice) as a disease model with high etiological validity. In a series of behavioral analyses, the mutant mice exhibit autistic-like behaviors such as impairments in sociality, flexibility, and working memory, demonstrating their face validity as an ASD model. To investigate the molecular basis of the observed abnormalities, we performed a transcriptomic analysis of their bulk adult brains and found that ASD risk genes were specifically enriched in the upregulated differentially expressed genes (DEGs), whereas KMT2C peaks detected by ChIP-seq were significantly co-localized with the downregulated genes, suggesting an important role of putative indirect effects of *Kmt2c* haploinsufficiency. We further performed single-cell RNA sequencing of newborn mouse brains to obtain cell type-resolved insights at an earlier stage. By integrating findings from ASD exome sequencing, genome-wide association, and postmortem brain studies to characterize DEGs in each cell cluster, we found strong ASD-associated transcriptomic changes in radial glia and immature neurons with no obvious bias toward upregulated or downregulated DEGs. On the other hand, there was no significant gross change in the cellular composition. Lastly, we explored potential therapeutic agents and demonstrate that vafidemstat, a lysine-specific histone demethylase 1 (LSD1) inhibitor that was effective in other models of neuropsychiatric/neurodevelopmental disorders, ameliorates impairments in sociality but not working memory in adult *Kmt2c*^+/fs^ mice. Intriguingly, the administration of vafidemstat was shown to alter the vast majority of DEGs in the direction to normalize the transcriptomic abnormalities in the mutant mice (94.3 and 82.5% of the significant upregulated and downregulated DEGs, respectively, *P* < 2.2 × 10^−16^, binomial test), which could be the molecular mechanism underlying the behavioral rescuing. In summary, our study expands the repertoire of ASD models with high etiological and face validity, elucidates the cell-type resolved molecular alterations due to *Kmt2c* haploinsufficiency, and demonstrates the efficacy of an LSD1 inhibitor that might be generalizable to multiple categories of psychiatric disorders along with a better understanding of its presumed mechanisms of action.

## Introduction

Autism spectrum disorder (ASD) is a group of neurodevelopmental conditions characterized by impaired sociality and repetitive behaviors [1–5], which is common and ~2.8% of children are diagnosed with this disorder in the United States [6]. Genetic factors are known to play critical roles in the pathogenesis of ASD, and recent large-scale human genetics studies have reported a number of genes robustly associated with ASD [7–10]. Specifically, the largest rare variant study to date involving 14,261 ASD families, 20,627 cases, and 42,610 controls has identified 185 ASD-associated genes at Benjamini-Hochberg-corrected *P* < 0.05, and the gene ontology (GO) term most significantly enriched among them was chromatin organization (Benjamini-Hochberg-corrected *P* value = 1.16 × 10^−19^, see Materials and methods for details). Therefore, the regulation of chromatin modification and gene transcription, which is closely related to the chromatin states, is considered the key biological process in the pathogenesis of ASD.

Among many genes involved in this pathway, *KMT2C* (*Lysine methyltransferase 2c*), encoding a catalytic unit of the histone methyltransferase complex regulating mono- and di-methylation of histone H3 lysine 4 (H3K4me1 and H3K4me2), is reported as one of the genes causally associated with ASD and neurodevelopmental disorders (NDD). A recent large-scale exome sequencing study identified significant enrichment of rare heterozygous loss-of-function (LOF) variants of *KMT2C* in ASD, and this gene is included in the list of most statistically robust 72 genes that passed a stringent exome-wide multiple testing correction [10]. De novo LOF variants of *KMT2C* were also identified in patients clinically diagnosed with Kleefstra syndrome, whose characteristic symptoms include autistic-like features [11]. Besides, other members of the H3K4 methylation regulators were reported to be associated with other neurodevelopmental syndromes with ASD symptoms and neuropsychiatric disorders such as schizophrenia [12–15]. Therefore, *KMT2C* haploinsufficiency is an established causative risk factor of ASD and NDD with autistic symptoms, and it has been indicated that H3K4 methylation plays an important role in brain development and cognitive functions [16]; however, little is known about how it causes neurodevelopmental deficits and how these conditions can be treated.

To address this, we established genetically modified mice modeling the *Kmt2c* haploinsufficiency (*Kmt2c*^+/fs^ mice) as a high etiological validity model of ASD/NDD and analyzed their behaviors such as sociality and flexibility to verify their face validity. To explore the molecular pathogenesis of ASD due to the loss of *KMT2C*, we analyzed the transcriptomic states in the brain by bulk or single-cell RNA sequencing (scRNA-seq). Finally, we tested the therapeutic effects of vifidemstat, an inhibitor of LSD1 (lysine-specific histone demethylase 1A), which may counteract *Kmt2c* haploinsufficiency, and demonstrated that this drug rescues the social deficits and the transcriptomic alterations in *Kmt2c*^+/fs^ mice.

## Materials and methods

### Establishment of *Kmt2c*^+/fs^ mice

To obtain single guide RNA (sgRNA) for the recognition of the *Kmt2c* locus, oligomers were first generated by annealing of the following primers: forward primer, 5′-CACCGGAATGGACCACCACCTCGAG-3′, and the reverse primer, 5′-AAACCTCGAGGTGGTGGTCCATTCC-3′. The oligomers were integrated into the BbsI site downstream of the U6 promoter of the pSpCas9(BB)-2A-GFP vector (PX458) (Addgene, MA, USA). This integration step followed our previous reports [17, 18]. After the integration, the sgRNA for the microinjection into the fertilized eggs was transcribed by MEGAshortscript Kit (Life Technologies, Carlsbad, CA, USA) using the generated sgRNA-SpCas9-GFP all-in-one vector as a template. A mixture of sgRNA (50 ng/μl) and Cas9 mRNA (100 ng/μl, Merck Millipore, MA, USA) was microinjected into the cytoplasm of fertilized eggs obtained from C57BL6/N mice (CLEA Japan, Inc., Tokyo, Japan). The injected eggs were transferred into the oviducts of pseudopregnant ICR female mice. Whole exome sequencing was performed to screen for possible off-target mutations using genomic DNA purified from tail samples of F1 mutant mice with the GenElute Mammalian Miniprep kit (Merck Millipore). We validated the introduction of novel insertion/deletion of nucleotides after the filtering as previously reported [18].

### Mouse housing and genotyping

The experimental protocols were approved by the Wako Animal Experiment Committee of RIKEN. *Kmt2c* heterozygous mutant mice were maintained by breeding *Kmt2c*^+/fs^ mice with wildtype C57BL6/N mice (CLEA Japan, Inc.) in a 12 h:12 h light-dark cycle. The mice for behavioral tests were obtained by in vitro fertilization using sperms of the heterozygous mutant mice and eggs derived from C57BL6/N mice (CLEA Japan, Inc.). Genotyping of all mutant mice was performed using genomic DNA derived from tail tips as previously reported [18]. Briefly, tail biopsies were conducted on postnatal days 14–21, and each tail tip was incubated in 100 μL of lysis buffer (25 mM NaOH, 0.2 mM EDTA) at 95 °C for 20–30 min. An equal volume of 40 mM TRIZMA hydrochloride was added to the lysate after incubation and the mixture was vortexed briefly. Afterward, 1 μL of the mixture was diluted with water 2.5-fold and was PCR-amplified under the conditions shown in Supplementary Table 1. The PCR products derived from wildtype and mutant allelles were discriminated by agarose gel electrophoresis or Sanger sequencing by an ABI 3730xl sequencer (Life Technologies, CA, USA) using BigDye Terminator V3.1 (Thermo Fisher Scientific, MA, USA) (Supplementary Table 1).

### qPCR

Total RNA was extracted from the hippocampal samples of 16 weeks old male mice with TRIzol RNA Isolation Reagents (Thermo Fisher Scientific). After DNase I treatment, reverse transcription was performed by SuperScript^TM^ III First-Strand Synthesis System (Invitrogen, MA, USA) using random hexamers. RT-qPCR was conducted using TB Green Premix Ex Taq^TM^ II (Takara Bio Inc., Shiga, Japan). The sequences of the primers are shown in Supplementary Table 1. The primers for *Kmt2c* were designed to recognize the full-length isoform of *Kmt2c*.

### Western blot of KMT2C

Nucleic and cytoplasmic protein samples were extracted from whole brains dissected from the mouse embryos at E15.5 using NE-PER Nuclear and Cytoplasmic Extraction Reagents (Thermo Fisher Scientific). The amount of protein in each sample was quantified by the Micro BCA^TM^ Protein Assay kit (Thermo Fisher Scientific), and 13 μg of proteins were separated using SDS-polyacrylamide gel electrophoresis (SDS-PAGE) with 3–8% NuPAGE Tris-Acetate Gels and NuPAGE Tris-Acetate SDS running buffer (Thermo Fisher Scientific). The separated proteins were transferred to the PVDF membrane under the conditions of 30 V, 4 °C for 18 h. The transferred membranes were blocked with 5% (for KMT2C) or 3% (for LaminA/C) skim milk in Tris-buffered saline (TBS) with 0.05% Tween-20 (TBST) for 30 min at 20–25 °C, then incubated overnight at 4 °C with rabbit anti-KMT2C (1:5000, ABE1851; Merck Millipore, Cambridge, UK) or rabbit anti-LaminA/C (1:1000, #2032; Cell Signaling Technology, Inc., MA, USA) primary antibody diluted by the corresponding blocking buffers. After washing five times with TBST for 5 min each, the membranes were incubated for 1 h with horseradish peroxidase (HRP)-conjugated anti-rabbit-IgG for anti-KMT2C or anti-LaminA/C (1:10000, sc-2357; Santa Cruz Biotechnology, TX, USA) at 20–25 °C. The immunoreactive bands were visualized using Amersham ECL Prime (GE Healthcare, Buckinghamshire, UK) and scanned using a LAS-3000 image analyzer (Fujifilm, Tokyo, Japan). The intensity of signals identified by each antibody was quantified by Fiji [19] (v.2.1.051).

### Behavioral tests

Details of all behavioral tests are described in the Supplementary Method. The major procedures are outlined below. We analyzed 15 male mice per condition in each behavioral test except for the IntelliCage analysis. The behavioral tests started after 13 weeks old. In the open field test, Y-maze test, Crawley’s three-chamber social interaction test, and prepulse inhibition (PPI) test, we combined the data from two batches of the experiments, of which one batch is from an analysis of untreated mice in the pharmacological experiments, in order to increase the statistical power.

#### Crawley’s three-chamber social interaction test

The social behavior of animals was measured in Crawley’s social interaction test chamber (O’HARA & CO., LTD., Tokyo, Japan). The chamber consisted of three areas separated by transparent walls with holes, and they could freely move three areas. On the first day, all stranger mice were habituated to the small cages in the arena for 10 min. On the second day, subject animals were first placed in the center area with a stranger caged mouse on one side, for 10 min (mouse cage vs. empty cage). The duration of the area staying was recorded.

#### Y-maze test

The Y-maze test was performed in an apparatus with three arms arranged at 120° intervals (O’HARA & CO., LTD) for 5 min. The alternation rate (the number to enter all three arms within three entries/[the total number of entries into arms] −2) was recorded.

#### Barnes maze test

In the Barnes maze test, twelve holes (40 mm in diameter) were evenly spaced around the circumference of a white circular arena (O’HARA & CO., LTD) and one escape box was set under the specific hole. The training session consisted of 20 trials (one or two trials/day, 5 min). After 1 day of the training sessions, probe tests were conducted.

#### Serial reversal test in IntelliCage analysis

The IntelliCages apparatus (39 × 58 × 21 cm; TSE systems, Inc. MO, USA) contains one chamber each at the four corners that is accessible through an open doorway. A radiofrequency identification transponder (Standard Microchip T-VA, DataMars, Lamone, Switzerland; and Trovan, Melton, UK) was implanted into the mouse dorso-cervical region under isoflurane inhalation anesthesia to track each mouse in the corner chambers. The light period was from 8:00 to 20:00 local time, and the dark period was from 20:00 to 8:00 (Light: Dark = 12 h:12 h). Behavioral tests using the IntelliCage system were conducted following the methods in the previous paper, and detailed procedures except for the serial reversal test are described in the Supplementary Method. The IntelliCage test was initiated when the mice are 16 weeks old. Following the completion of all other tests shown in Supplementary Fig. 1, the serial reversal test was started (*N* = 6, 40–41 weeks old). The serial reversal test within this system was performed as follows. Before the serial reversal test, one of the four corners (correct corner) is accessible to drinking water for seven days (place learning test). After the place learning test, the correct corner was changed to the diagonally opposite corner to investigate the reversal learning and train mice to learn the change of the correct corner (place learning reversal test). The serial reversal test was started after the place learning reversal test for five days. The correct corner was switched to the other diagonal line on the first day of the serial reversal test. Afterward, the correct corner was changed to the corner diagonally opposite to the corner of the previous day, every day, and this diagonal moving was continued for four days. These processes were repeated three times, and the rate of the visits to the correct corner on the previous day in the first 15 min was calculated as an indicator of inflexibility. During these tests, the drinking water was accessible from 21:00 to 23:59 local time.

### Sample preparations for RNA-seq of bulk tissues

Total RNAs were extracted from forebrain samples derived from 11 weeks old mice and vafidemstat- or DMSO-treated 22 weeks old mice using Trizol reagent (Thermo Fisher Scientific), followed by the treatment with DNase I (Takara Bio Inc.) to remove DNA. The quality of RNA samples was analyzed by Agilent Bioanalyzer (Agilent Technologies, Inc., CA, USA), and samples with RNA integrity number (RIN) > 7.9 were subjected to RNA-seq. The preparation of the library and sequencing were conducted by Novogene Co., Ltd, Beijing, China. The mRNA was enriched from total RNA using poly-T oligo-attached magnetic beads. After fragmentation, the first-strand cDNA was synthesized using random hexamer primers followed by the second-strand cDNA synthesis using NEBNext Ultra RNA Library Prep Kit for Illumina (New England BioLabs, MA, USA). Sequencing was performed on the Novaseq-6000 platform (Illumina) following the manufacturer’s instructions. The amount of data per sample was ~6 Gb (~20 M of 150 bp × 2 paired-end reads).

### Chromatin immunoprecipitation

Chromatin immunoprecipitation was performed as previously described [20]. Two 16 weeks old wildtype C57BL6/N mice were used to obtain data of biological replicates. The hippocampal tissue of each mouse was dissociated using a 100 μm cell strainer (SPL Life Sciences Co., Ltd., Gyeonggi-do, Korea) with a syringe plunger in 4 mL of ice-cold PBS, and further homogenized using a Dounce tissue grinder (Sigma-Aldrich) with a small clearance pestle (pestle B) for 10 strokes and centrifuged at 1500 × g, 5 min, 4 °C. Fixation was performed by re-suspending the cell pellet in 1 mL of fixation buffer (PBS supplemented with 1% formaldehyde and 18.7 μM disuccinimidyl glutarate) and incubating at 25 °C for 10 min. The fixation was stopped by adding 100 μL of 2.5 M Glycine. The fixed cells were collected by centrifugation at 1500 × g, 4 °C for 5 min, and washed twice in PBS by re-suspending the pellet in 1 ml of PBS and centrifuging at 1500 × *g*, 4 °C for 5 min at each wash cycle. The fixed cell pellet was snap-frozen in liquid nitrogen and stored at −80 °C until use. The chromatin lysate was prepared by sonication with Covaris E220 (Covaris, MA, USA). Before the sonication, the stored pellet was washed once with 1 ml of lysis buffer 1 (50 mM HEPES-KOH, 140 mM NaCl, 1 mM EDTA, 10% (w/v) Glycerol, 0.5% (w/v) NP-40, 0.25% (w/v) Triton X-100, 0.1 × Protease Inhibitor Cocktail (Sigma)), once with 1 ml of lysis buffer 2 (10 mM Tris-HCl, 200 mM NaCl, 1 mM EDTA, 0.5 mM EGTA, 0.1 × Protease Inhibitor Cocktail) and twice with 1 ml of RIPA buffer (# 89900; Thermo Fisher Scientific) supplemented with 0.1 × Protease Inhibitor Cocktail. Each washing step was performed by re-suspending the pellet in the buffer and centrifuging at 2000 × *g*, 4 °C for 5 min. After the washing step, the cell pellet was re-suspended in 1 ml of RIPA buffer supplemented with 1 × Protease Inhibitor Cocktail and transferred to a milliTUBE (Covaris). The sonication condition of the Covaris E220 was PIP: 175 W, Cycles per Burst: 200, Duty Factor: 15%, for 30 min at 7 °C. After the sonication, the chromatin lysate was centrifuged at 20,000 × *g*, 4 °C for 5 min, and the supernatant was collected.

Immunoprecipitation was performed in RIPA buffer containing 0.5 mg/ml BSA (15561-020, Invitrogen), the chromatin lysate equivalent to 1.6–1.8 × 10^6^ cells, and 50 µl of Protein A beads coupled with 15 µg of anti-KMT2C antibody (ABE1851; Merck Millipore) for the duration of 4 h at 4 °C. After the reaction, the beads were washed five times in 1 ml of low salt buffer (20 mM Tris-HCl (pH 8.0), 0.1% SDS, 1% (w/v) Triton X-100, 2 mM EDTA, 150 mM NaCl), and three more times with 1 ml of high salt buffer (20 mM Tris-HCl (pH 8.0), 0.1% SDS, 1% (w/v) TritonX-100, 2 mM EDTA, 500 mM NaCl). Washing of the beads was performed by placing the tube on the DynaMag 2 magnet (Thermo Fisher Scientific), removing the supernatant, and re-suspending the beads in the next buffer. The chromatin complex was eluted from the magnetic beads by agitation in 200 µl of ChIP elution buffer (10 mM Tris-HCl (pH 8.0), 300 mM NaCl, 5 mM EDTA, 1% SDS) for 15 min at room temperature. On the magnet, the supernatant containing the chromatin complex was collected, transferred to a new tube, and incubated at 65 °C, overnight, for reverse-crosslinking. The chromatin complex was further incubated with 50 µg/ml RNase A for 20 min at 37 and with 0.5 mg/ml Proteinase K at 55 °C for 40 min. ChIP DNA was purified by phenol-chloroform extraction followed by ethanol precipitation. Input DNA was extracted from 20 µl of the ChIP lysate in the same way as the ChIP DNA. Libraries were prepared using 10 ng of input DNA and 0.75–1.0 ng of ChIP DNA with the KAPA LTP Library Preparation Kit (# KK8232; KAPA Biosystems, MA, USA) and UDI adapters (NOVA-514180, BIOO Scientific, TX, USA). Sequencing was performed on the HiSeq 1500 platform (Illumina) to obtain ~40 M single-end 80 bp reads.

### Sample preparations for microarray analysis

Total RNAs were extracted from hippocampal samples of 16 weeks old mice using the same method as for bulk RNA-seq and then treated with DNase I. A microarray analysis was performed by using the Clariom^TM^ D Assay, mouse (902513, Thermo Fisher Scientific) platform according to the manufacturer’s protocol. The library preparation and assay were performed at RIKEN Center for Brain Science, Research Resource Division (RIKEN CBS RRD).

### Quartz-seq2

We performed a scRNA-seq analysis using Quartz-seq2 according to the protocol described by developer [21], as detailed below.

#### Single-cell separation

Neonatal forebrain (prosencephalon) samples were dissected from P4 mouse pups under a stereomicroscope and temporarily stored in ice-cold Hank’s balanced salt solution. The separation to single cells was performed using Pierce Primary Neuron Isolation Kit (Thermo Fisher Scientific) according to the manufacturer’s protocol. Tail samples were used for genotyping of the *Kmt2c* frameshift mutation and verification of sex by amplification of *Sry* and *Kdm5d* fragments on the Y chromosome. The PCR conditions are shown in Supplementary Table 1. The separated single cells were stained by 7-AAD to exclude the dead cells. These cells were analyzed using flow cytometry FACSAria II Special Order System (BD Bioscience, CA, USA) with 100 μm nozzles. Single cells were isolated into 384-well PCR plates with 1 μL of lysis buffer (0.1111 μM respective RT primers (v32_384p01, unique molecular identifier (UMI) sequence -cell barcode -oligo dT), 0.12 mM dNTP mix, 0.3% NP-40, 1 unit/μL RNasin plus) in each well (Supplementary Fig. 2a, b). During single-cell sorting, 384-well PCR plates were kept on a 384 aluminum stand at 4 °C using CoolRack XT PCR384 (Corning, NY, USA). The 384-well PCR plates were sealed by the PX1 PCR Plate Sealer (BIO-RAD, CA, USA) and centrifuged at 10,000 × *g* and 4 °C for 1 min immediately after the cell collection. We agitated the plates at 2600 rpm and 4 °C for 1 min using ThermoMixer C (Eppendorf, Hamburg, Germany) followed by another centrifugation. The prepared 384-well plates were immediately cryopreserved at −80 °C and maintained until subsequent RT for cell barcoding.

#### Whole transcript amplification

Whole-transcript amplification was performed by using the 384-well PCR plates prepared above. The cryopreserved 384-well plates with single-cell lysates were centrifuged at 10,000 × *g* and 4 °C for 1 min, followed by denaturation of total RNA in each 384-plate at 70 °C for 90 s and hybridization of RT primers to poly-adenylated RNA at 35 °C for 15 s using the Dual 384-Well GeneAmp^TM^ PCR System 9700. We removed the seal and added 1 μL of RT premix (2× Thermopol buffer, 1.25 units/μL SuperScript III, 0.1375 units/μL RNasin plus) to 1 μL of lysis buffer in each well on the 384 aluminum plate. We agitated the re-sealed plates at 2600 rpm and 4 °C for 1 min. The plates were then centrifuged at 10,000 × *g* and 4 °C for 1 min, and the reverse transcription (RT) was reacted at 35 °C for 5 min and 50 °C for 50 min. The RT was stopped at 70 °C for 15 min. We turned the plates upside down on the assembled collector and centrifuged the plates with an assembled collector at 3010 × *g* and 4 °C for 3 min using swing-bucket rotors. Subsequently, we collected the cDNA solution into a disposable reservoir. We purified and concentrated the cDNA solution using the DNA Clean & Concentrator^TM^-5 kit (Zymo Research, CA, USA) and extracted it with 20 μL of nuclease-free water. We added 25 μL of TdT solution (1× Thermopol buffer, 2.4 mM dATP, 0.0384 units/μL RNase H (Invitrogen), 26.88 units/μL terminal transferase (Roche, Basel, Switzerland)) into 20 μL of extracted cDNA. A poly(A) tailing was reacted at 37 °C for 75 s followed by the inactivation at 65 °C for 10 min. After the dispersion of ~11 μL of solution into four wells from 45 μL of TdT solution, 46.16 μL of PCR I premix (1.08492× MightyAmp Buffer version 2, 0.06932 μM Tagging primer, 0.05415 units/μL MightyAmp DNA polymerase (Takara Bio Inc.)) was added to 11 μL of TdT solution for the respective wells of the PCR tube. The solution was mixed at 2000 rpm and 4 °C for 2 min. We denatured the solution at 98 °C for 130 s and hybridized tagging primer to poly(A)-tailed cDNA at 40 °C for 1 min. After that, the Increment step by heating to 68 °C at 0.2 °C every second and second-strand synthesis at 68 °C for 5 min were conducted. 50.232 μL of PCR II premix (0.99697× MightyAmp Buffer version. 2, 1.8952 μM gM primer) was mixed with 56.16 μL of PCR I solution. Subsequently, the cDNA was amplified by 12 cycles with the following conditions: 98 °C for 10 s, 65 °C for 15 s, and 68 °C for 5 min. Finally, all of the PCR solutions derived from one 384-well PCR plate were transferred to a 15 mL polypropylene centrifuge tube. 32.1 μL of 3 M sodium acetate (pH 5.2) and 6.42 mL of PB-Buffer (QIAGEN, Düsseldorf, Germany) were added to the PCR solution. The mixture was purified by a MinElute Spin Column (QIAGEN). Purified cDNA was extracted into 40 μL of nuclease-free water. The extracted cDNA was additionally purified with 26 μL of Ampure XP beads.

#### Preparation of sequence library and sequencing

5–10 ng of the amplified cDNA in 130 µL of nuclease-free water was fragmented using an M220 Focused-ultrasonicator (Covaris) under the following conditions: duty factor 10.0, peak power 50.0, cycles per burst 200, and treatment time 65 s. The fragmented cDNA was purified using the DNA Clean & Concentrator^TM^-5 kit. Purified cDNA was extracted into 10 μL of nuclease-free water. We then added 2 μL of End-Repair premix (1.4 μL of End repair & A-tailing buffer and 0.6 μL of End repair & A-tailing Enzyme (KAPA Biosystems)) to 10 μL of fragmented cDNA solution followed by the incubation at 37 °C for 60 min and 65 °C for 30 min. 2 μL of adapter buffer (1.5 μM truncated adapter, 10 mM Tris-HCl pH 7.8, 0.1 mM EDTA pH 8.0, 50 mM NaCl) and 8 μL of ligation premix (6 μL of ligation buffer, 2 μL of DNA ligase (KAPA Biosystems)) was added at 4 °C. After that, adapters were ligated at 20 °C for 15 min. After the ligation, 18 μL of Ampure XP beads was added to 22 μL of adapter ligation solution. Adapter-ligated cDNA was extracted into 20 μL of nuclease-free water. The cDNA mixtures were mixed with 32 μL of PCR premix (25 μL of 2 × KAPA HiFi ready mix, 1.75 μL of 10 μM TPC2 primer (HPLC-purified), 10 μM P5-gMac hybrid primer (HPLC-purified)) to 18 μL of adapter-ligated cDNA. The solution was denatured at 98 °C for 45 s. Subsequently, cDNA was amplified by eight cycles under the following conditions: 98 °C for 15 s, 60 °C for 30 s, and 72 °C for 1 min. 40 μL of Ampure XP beads was added to 50 μL of PCR solution. Purified sequence-library DNA was eluted into 20–30 μL of nuclease-free water. The DNA concentration and DNA size of sequence library DNA were checked using KAPA Library Quantification Kits (NIPPON Genetics, Tokyo, Japan). The sequencing was performed by RIKEN GENESIS CO., LTD. (Japan) using NextSeq (Illumina, CA, USA). The lengths of reads were 23 bp (Read1) and 63 bp (Read2).

### Vafidemstat preparation and administration

In the pharmacological experiment with an LSD1 inhibitor, we utilized vafidemstat (also known as ORY-2001, MedChemExpress, NA, USA). Vafidemstat was dissolved in dimethyl-sulfoxide (DMSO) to prepare the stock solution (50 mg/mL). The stock solution was stored at −80 °C until use. Vafidemstat was administered via drinking water according to a previous report [22]. Before the experiment, we measured that 5 mL per day of water was taken by a mouse on average. We calculated the final concentration in the drinking water assuming the weight of a mouse to be 35 g. To administer 0.96 mg/kg day of vafidemstat to a mouse via drinking water, the stock solution was diluted by water to 6.72 μg/mL. The drinking water was changed every week. Vafidemstat was administered to 15 male mice per condition for 4 weeks before the behavioral tests. The data on the structure was downloaded from ChemSpider (http://www.chemspider.com/), and the structural formula was drawn using the ChemSketch software (ACD/Labs, Ontario, Canada).

### Data analysis

#### RNA-seq of bulk tissues

Quality control of the fastq files obtained by the above-described experimental procedures was performed using FASTQC (v.0.11.9) (https://www.bioinformatics.babraham.ac.uk/projects/fastqc/). Read mapping on the mm10 mouse genome was performed using STAR [23] (v.2.7.9a) with “--outFilterMultimapNmax 1” after removal of the adapter sequence by trim_galore [24] (v.0.6.6) with default settings. The output SAM files were converted to BAM files by samtools [25] (v.1.3.1). Ribosomal RNAs were removed after the mapping using “intersectBed” functions of bedtools [26] (v.2.30.0). The reference BED file of ribosomal RNAs based on GRCm38/mm10 was obtained from UCSC Genome Browser (https://genome.ucsc.edu/). Read counts of each gene in the filtered BAM files were calculated using featureCounts [27] (v.2.0.1). The gene annotation was based on the gtf file of GRCm38 (Ensembl release 102) derived from https://ftp.ensembl.org/pub/release-102/gtf/mus_musculus/. After extracting protein-coding genes using Biomart (GRCm38.p6) as a reference, the read counts of genes were normalized, and the differentially expressed genes (DEGs, uncorrected *P* value < 0.05) were identified using DESeq2 [28] (v.1.30.1) in R (v.4.0.4). Mitochondrial genes and genes with baseMean < = 20 were excluded from the downstream analysis. We analyzed four mice per genotype in the analysis of adult forebrain and three mice per conditions in the pharmacological experiment. We also verified the genotypes of mice by confirming the presence or absence of reads harboring the *Kmt2c* frameshift valiant using the Integrative Genomics Viewer (IGV) [29]. We note that the data from one *Kmt2c*^+/fs^ mouse treated with DMSO failed our quality control criteria and was excluded from the downstream analyses.

#### Overlap between DEGs in Kmt2c^+/fs^ mice and ASD exome genes or ASD postmortem brain DEGs

To analyze the overlap of DEGs in *Kmt2c*^+/fs^ mice with ASD-associated genes, the gene symbols in mice were converted to the orthologous genes in humans by the following procedures. To obtain a correspondence table of orthologous genes between mice and humans, we first utilized the “getLDS” function of the biomaRt package [30, 31] (v.2.46.3) in R (v.4.0.4). We retrieved the orthologous relationships between murine gene symbols, human gene symbols, and human Ensembl gene IDs. In the obtained list, there were cases where a particular murine gene was converted to multiple human genes or multiple murine genes corresponded to a single human gene. In such cases, we selected the human gene that exactly matched the capitalized mouse gene symbol. We then assigned human Ensembl gene IDs to each mouse gene based on the generated correspondence table (“Humanized ENSGID” in Supplementary Tables 2 and 3). Subsequently, we examined the overlap between DEGs in *Kmt2c*^+/fs^ mice and the 185 ASD exome genes (see Results for details) or DEGs in human ASD postmortem brains, which were obtained from ref. [10] and ref. [32], respectively, based on the human Ensembl gene IDs. In this analysis, we focused on protein-coding genes in the nuclear genome. The overlaps were statistically evaluated by a hypergeometric test using the phyper function in R (v.4.0.4). The background gene lists in each test were defined as those that were detected in each bulk RNA-seq or scRNA-seq analysis and successfully converted to human Ensembl gene IDs.

#### Linkage disequilibrium score regression (LDSC)

We analyzed the enrichment of single nucleotide polymorphisms (SNP)-based heritability in DEGs in *Kmt2c*^+/fs^ mice using LDSC (v.1.0.1). After the conversion of the identified DEGs into human Ensemble gene IDs as described above, we performed an LDSC analysis using significant DEGs (uncorrected *P* < 0.05) and the top 1000 and top 2000 DEGs (sorted by uncorrected *P* values) identified by RNA-seq of bulk adult mouse forebrains, and the top 1000 DEGs in each cell cluster identified by scRNA-seq of neonatal forebrains. The summary statistics in ASD genome-wide association studies (GWAS) meta-analysis was downloaded from https://bitbucket.org/steinlabunc/spark_asd_sumstats/src/master/ASD_SPARK_iPSYCH_PGC.tsv.gz. This GWAS summary statistics file was formatted for LDSC using the “munge_sumstat” function in LDSC. Analyses of partitioned heritability of the genes of interest (i.e., DEGs) were performed using the baseline model (v2.2) of 97 genomic annotations and the data of variant frequencies (1000G_Phase3_frq), genotypes (1000G_EUR_Phase3_plink), and weights (1000G_Phase3_weights_hm3_no_MHC) from the 1000 Genomes Project Phase 3.

#### Chromatin immunoprecipitation followed by sequencing (ChIP-seq)

Removal and trimming of the illumina adapter sequences from the fastq file were performed by trim_galore (v.0.6.4) with the default settings. Mapping of the sequencing reads to the mm10 mouse genome was performed by Bowtie2 [33] (v.2.3.5.1). Peak-calling was performed by MACS2 [34] (v.2.1.1.20160309). The KMT2C peaks were defined as the common peaks among the identified peak sets derived from two biological replicates (rep1 and rep2) and merged read data of the duplicates (rep1 + rep2). The common peaks were extracted using the BED files of each data by “intersectBed” functions of bedtools. The peaks of KMT2C were annotated by HOMER (v.4.11). The fastq files for H3K4me1 and H3K4me3 ChIP-seq from a previous study were obtained from GEO (accession number: GSE85873). Read mapping was performed using the same method as for KMT2C ChIP-seq. The BAM files derived from H3K4me1 and H3K4me3 ChIP-seq (8 weeks old, male mice with B6NCrl background) of cortical plates were obtained from the ENCODE project (H3K4me1 ENCFF438JYD and ENCFF151JWT; H3K4me3, ENCFF474SND, and ENCFF892POZ). Two BAM files for each histone mark were merged by the “merge” option of samtools [25] (v.1.3.1). The heatmaps were plotted by the functions of “computeMatrix” and “plotHeatmap” of deepTools [35] (v.3.3.0) using the BED files of KMT2C peaks as reference. For the analyses of genes bound by KMT2C in their promotor-TSS regions (KMT2C target genes), only the protein-coding genes annotated by Biomart based on the mm10 mouse genome were included. The BED file of SETD1A peaks of cortical samples was obtained from a previous study [13]. The SETD1A peaks were annotated by HOMER (v.4.11) to define the SETD1A target genes as we did for KMT2C. The overlap between KMT2C target genes and DEGs or SETD1A target genes was statistically assessed by a hypergeometric test.

#### Microarray

The CEL files were analyzed using the GeneSpring software (Agilent Biotechnologies). The SST-RMA method was utilized for the normalization of the expression signals. Genes with uncorrected *P* < 0.05 calculated by Mann-Whitney’s test and fold change > 1.1 were defined as DEGs. The overlap between the protein-coding DEGs and the KMT2C target genes was analyzed as described above.

#### scRNA-seq

To generate a gene-cell UMI matrix, the BCL files from an Illumina sequencer were analyzed by the Q2-pipeline (https://github.com/rikenbit/Q2-pipeline) provided by Nikaido lab that developed Quartz-seq2. Subsequent analyses of the UMI matrix were performed by using Seurat [36] (v.4.0.2) in R (v.4.0.4). Cells with the following conditions were filtered out as outliers; 1) the number of detected genes in the cell, that is nFeature_RNA (i.e., gene count per cell), is less than 2500 or more than 10,000, 2) the proportion of mitochondrial UMI is greater than or equal to 12%. After the above quality check (Supplementary Fig. 2c-i), normalization of the expression abundance and extraction of highly variable features for the clustering and scaling were performed with the default settings. For the dimensionality reduction, principal components (PCs) were extracted by the “JackStraw” and “ScoreJackStraw” functions, and the top 15 PCs were utilized for non-linear dimensionality reduction based on the uniform manifold approximation and projection (UMAP) method. Marker genes of each cell cluster were identified by the “FindAllMarkers” function with the default settings. Cell clusters were manually annotated based on the expression patterns of general cell type markers. DEGs in each cluster were identified using the “FindMarkers” function specifying test.use = MAST. The parameter for the logfc.threshold was set as 0.0001. Genes with uncorrected *P* value < 0.05 were considered DEGs. In the analysis of the cellular composition in the *Kmt2c*^+/fs^ and *Kmt2c*^+/+^ mice, we performed a 2 × 18 (genotypes × cell clusters) Fisher’s exact test using the “fisher.test” function in R (v.4.0.4) with the default setting except for the method parameter set to “simulate.*p*.value = TRUE”.

#### Analysis of cell clusters with ASD-associated transcriptomic alterations

To characterize the DEGs in each cell cluster mainly in the context of ASD-associated gene enrichment, we performed the following four analyses.

1. After identifying DEGs (uncorrected *P* < 0.05) in each cell cluster, we evaluated the difference between the observed DEG count and the expected number of DEGs estimated from the number of cells in the cluster by a residual analysis. Because the number of cells in each cluster correlates with the number of DEGs, we employed this method rather than simply counting the number of DEGs in each cluster. The expected number of DEGs in each cluster was estimated by drawing the regression line of the number of cells and DEGs in the 18 clusters. The standardized residuals were obtained by the “chisq.test” function in R (v.4.0.4) using the cross-tabulation table of the observed and expected number of DEGs. The standardized residuals were then converted into the one-tailed *P* value using the “pnorm” function in R (v.4.0.4).
2. We analyzed the overlap between the DEGs in each cell cluster and the 185 ASD exome genes in ref. [10] by a hypergeometric test using the method described in the “Overlap between DEGs in *Kmt2c*^+/fs^ mice and ASD exome genes or ASD postmortem brain DEGs” section.
3. We analyzed if the top 1000 DEGs in each cell cluster are enriched for the SNP-based ASD heritability [37] by LDSC [38, 39], as described in the “Linkage disequilibrium score regression (LDSC)” section. This threshold for DEG selection was determined according to the results of the RNA-seq analysis of bulk forebrain tissues.
4. We analyzed the overlap between the DEGs in each cell cluster and the DEGs in a study of ASD postmortem brains (Benjamini-Hochberg corrected *P* < 0.05) [32] by a hypergeometric test, as in (2).

We then ranked the cell clusters according to the *P* values obtained by each analysis, calculated the sum of the ranks in the four analyses for each cluster, and considered clusters with a smaller rank-sum to have a higher ASD-associated transcriptomic alteration score. When two or more clusters showed the same total scores, we ranked them using the combined *P* value of the four *P* values calculated by Fisher’s method using the “metap” package (v.1.8) in R (v.4.0.4). These analyses were performed for all, upregulated, and downregulated DEGs. To assess the correlation of ranks between the analyses of upregulated and downregulated DEGs, Spearman’s rank correlation coefficient (ρ) was calculated using the “cor.test” function in R (v.4.0.4). The method parameter was set to “spearman”.

#### Pseudotime trajectory analysis

Pseudotime trajectory analysis was performed by Monocle3 (v.1.2.7) in R (v.4.1.3) using the UMI matrix including neuronal, astrocyte, and oligodendrocyte cells (cluster 0, 1, 2, 3, 5, 7, 8, 9, 11, 12, 14, 16, and 17) as the input. The top 10 PCs were utilized for the normalization of the data. The dimensionality reduction was performed by the default setting of the UMAP method. Pseudotime trajectories were generated by the “learn_graph” and “order_cells” functions with the default settings. The root node of the trajectory graph was manually selected.

#### Gene ontology enrichment analysis

We used Metascape [40] (https://metascape.org/) for gene ontology enrichment analyses of genesets of interest. The parameters in “Pathway & Process Enrichment” were set with the default settings (Min Overlap, 3; *P* Value Cutoff, 0.01; Min Enrichment, 1.5). “GO Molecular Functions”, “GO Biological Processes” and “GO Cellular Components” were selected as the input of the terms of the pathways. We performed the analyses based on Ensemble gene IDs. The reference protein-coding gene lists were obtained from Biomart. Mitochondrial genes were excluded from the analysis. We used the mm10 mouse and hg38 human genomes as references. The output files generated by Metascape were subjected to network visualization of the GO terms with Benjamini-Hochberg-corrected *P* value < 0.05 by Cytoscape [41] (v.3.9.1). GO networks were manually annotated.

#### Downstream analysis of RNA-seq data obtained after administrating vafidemstat or vehicle

To evaluate the direction of the effect of vafidemstat, a binomial test was performed using the “binom.test” function in R (v.4.0.4) with the default settings and the theoretical success rate = 0.5. Pearson’s correlation coefficients (*r*) of the fold changes shown in the pharmacological experiments were calculated using the “cor.test” function in R (v.4.0.4) with the default settings. The method parameter was set to “pearson”. For the comparison of two correlation coefficients, we employed the method proposed by Steiger [42]. This comparison was performed using the “cocor” package [43] (v.1.1–4) in R utilizing the option for a comparison of two overlapping correlations based on dependent groups. We note that genes with baseMean < = 20 were excluded from these analyses.

### Statistical analyses

All statistical details are indicated in the Materials and methods and/or in the figure legends. The statistical analyses, if not otherwise specified, were performed using R (v.4.0.4).

## Results

### *Kmt2c*^+/fs^ mice exhibit ASD-like phenotypes

**Fig. 1:**
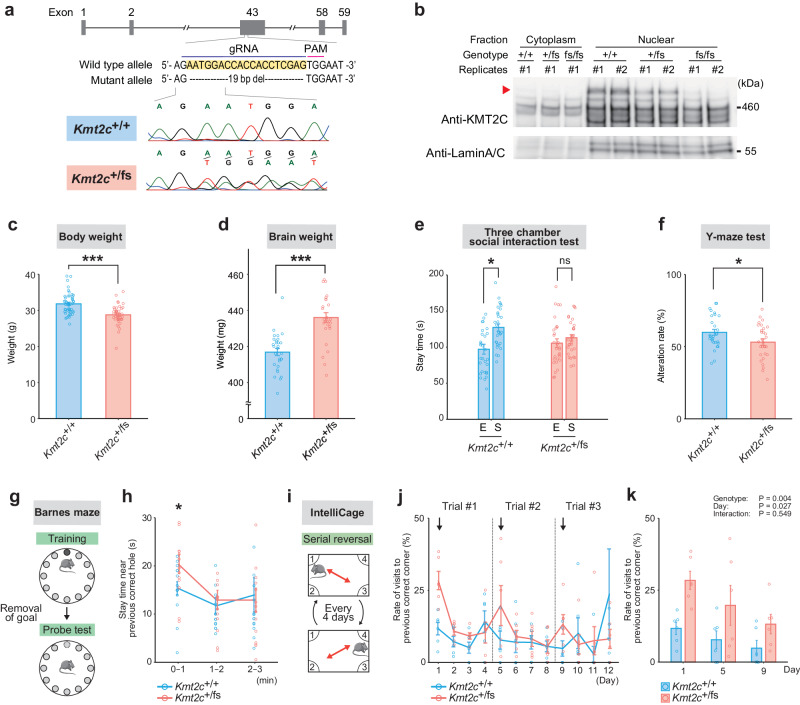
Generation of *Kmt2c*^+/fs^ mice and their ASD-like behaviors. **a** Schematic diagram of the mutation introduced in *Kmt2c*^+/fs^ mutant mouse line. The 19 bp deletion was introduced by the CRISPR/Cas9 system. **b** Western blot analysis of KMT2C using anti-KMT2C antibody. Proteins of the nucleic fraction were extracted from the whole brain samples derived from murine embryos at E15.5. The red arrowhead indicates the full-length KMT2C protein. **c** Measurement of the body weights. The *Kmt2c*^+/fs^ mice show significantly lower body weights (*Kmt2c*^+/+^, N = 47; *Kmt2c*^+/fs^, N = 41, 12 weeks old). ***P < 0.001. **d** Measurement of the brain weights. The weights of the whole brains of *Kmt2c*^+/fs^ mice are significantly higher (*Kmt2c*^+/+^, N = 29; *Kmt2c*^+/fs^, N = 28, 11 − 13 weeks old). ***P < 0.001. **e** Three-chamber social interaction test. The wildtype *Kmt2c*^+/+^ mice stay significantly longer in the chamber with a stranger mouse (S) than in the empty chamber (E), whereas *Kmt2c*^+/fs^ mice do not show a significant difference (*Kmt2c*^+/+^, N = 30; *Kmt2c*^+/fs^, N = 30, 16 − 21 weeks old). *P < 0.05. **f** Y-maze test. The alteration rates of *Kmt2c*^+/fs^ mice are significantly lower. (*Kmt2c*^+/+^, N = 30; *Kmt2c*^+/fs^, N = 30, 16 − 21 weeks old). *P < 0.05. **g** Schematic diagram of the Barnes maze test. After the training session of the Barnes maze to learn the goal among the twelve holes (upper image, the goal is schematically shown as a black circle. Results are shown in supplementary fig. 5j), the behaviors of mice immediately after the removal of the goal box were evaluated (probe test, lower image, the removed goal [the previous correct hole] is shown as the dashed circle). **h** Stay time near the previous correct hole in the probe test in the Barnes maze test. The *Kmt2c*^+/fs^ mice stay significantly longer near the previous correct hole (dashed circle in lower image of (**g**)) in the first minute of the probe test in the Barnes maze test (*Kmt2c*^+/+^, *N* = 15; *Kmt2c*^+/fs^, N = 15, 14 − 22 weeks old). *P < 0.05. **i** Schematic diagram of the serial reversal test in the IntelliCage analysis. In this test, one of the four corners (correct corner) is accessible to drinking water, and the correct corner is changed to the diagonally opposite corner every day. The diagonal line of the change of the correct corner is also switched every four days (i.e., days 1, 5, and 9 are the days of a rule change). **j**, **k** Rates of the visits to the previously correct corner in the IntelliCage serial reversal test on each day (**j**) and on the days of the rule change (**k**). The arrows in (**j**) indicate the days of the rule changes. On the days of the rule change (i.e., day 1, 5, and 9), the *Kmt2c*^+/fs^ mice significantly more frequently visit the incorrect corner which had been the correct corner one day before the day of the diagonal line change during the first 15 min of the test session (**k**) (*Kmt2c*^+/+^, N = 6; *Kmt2c*^+/fs^, N = 6, 40 − 41 weeks old). Two-way repeated measures ANOVA was used for the statistical comparison in panel (**k**). Genotype, *P* = 0.004; Day, *P* = 0.027; Interaction, *P* = 0.549. In these analyses except for (**k**), Welch’s t-test was used for the statistical comparison. The data are presented as mean ± standard error of the mean (SEM). We used male mice.

To investigate the consequence of *KMT2C* haploinsufficiency in a model organism, we introduced mutations in a canonical exon of *Kmt2c* of mice via non-homologous end joining (NHEJ) using the CRISPR/Cas9 system [44]. Among the generated founder mice with NHEJ mutations, we selected the mutant mice harboring a frameshift mutation in the *Kmt2c* gene and not carrying off-target insertion/deletion in the coding region (see Materials and methods for details), and established *Kmt2c* heterozygous mutant mice (*Kmt2c*^+/fs^ mice) carrying a 19 bp exonic deletion (Fig. 1a). The expression level of *Kmt2c* was significantly lower in *Kmt2c*^+/fs^ mice than in wildtype controls (Supplementary Fig. 3a). When we analyzed the expression of the KMT2C proteins using embryonic brain samples (E15.5), as homozygous knockout of *Kmt2c* is lethal at late embryonic stages, we observed that the signal of the full-length KMT2C was absent or very faint in *Kmt2c*^fs/fs^ mice with no detectable bands specific to the mutants (Fig. 1b). The intensity of the signals from the full-length KMT2C was significantly different across genotypes (Supplementary Fig. 3b, *P* = 1.55 × 10^−3^, one-way analysis of variance [ANOVA]). Despite *Kmt2c*^+/fs^ mice exhibiting lower body weight (Fig. 1c, *P* = 2.97 × 10^−6^, Welch’s t-test), the weight of the brain of *Kmt2c*^+/fs^ mice was significantly heavier than that of the wildtype mice (Fig. 1d and Supplementary Fig. 4, *P* = 5.07 × 10^−7^, Welch’s t-test), which resembles the brain overgrowth found in some ASD patients [45].

After confirming the validity of *Kmt2c*^+/fs^ mice as a haploinsufficiency model, we performed a behavioral test battery (Fig. 1e-h and Supplementary Fig. 5). In the three-chamber social interaction test, we revealed that *Kmt2c*^+/fs^ mice did not show a preference for the chamber with a stranger mouse (*P* = 0.316, Welch’s t-test), whereas wildtype mice showed a significant preference (*P* = 7.54 × 10^−4^, Welch’s t-test), indicating lower sociality in the mutants (Fig. 1e). The *Kmt2c*^+/fs^ mice exhibited lower alteration rates in the Y-maze test (*P* = 0.0269, Welch’s t-test), a sign of impaired spatial working memory (Fig. 1f). Although *Kmt2c*^+/fs^ mice showed no significant impairment of spatial learning in the Barnes maze test (Supplementary Fig. 5j), in which mice seek a goal box under one of the 12 holes, they stayed near the previous correct hole for a significantly longer time during the first minute after the removal of the goal in the probe test (Fig. 1g, h, *P* = 0.0432, Welch’s t-test). It suggests that *Kmt2c*^+/fs^ mice are less flexible to changes in circumstances. A similar behavioral change that could reflect a lack of flexibility in *Kmt2c*^+/fs^ mice was observed in the serial reversal task within the test battery performed by using the IntelliCage system [18, 46], an apparatus with which various behaviors can be automatically assessed. In the IntelliCage serial reversal test, mice can drink water only at one of the four corners and the correct corner is changed diagonally every day, with a change of the diagonal line every four days (Fig. 1i). In the first 15 min of drinking sessions on the days after diagonal line changes (i.e., days 1, 5, and 9), *Kmt2c*^+/fs^ mice exhibited significantly higher rates of incorrect visits to the corner which opened on the previous day (Fig. 1j, k, *P* = 1.30 × 10^−3^, Welch’s t-test), whereas no significance was observed in those on other days (*P* = 0.692, Welch’s t-test). In other behavioral tests using IntelliCage, *Kmt2c*^+/fs^ mice did not show impairments in spatial memory, impulsivity, attention, and delay discounting (Supplementary Fig. 1). Also, we observed no significant differences in the general health examination, open field, elevated plus maze, light-dark transition, rotarod, hotplate, Porsolt’s forced swim, tail suspension, and fear conditioning tests between *Kmt2c*^+/fs^ and wildtype mice, indicating that there were no prominent abnormalities in phenotypes related to basal activity, anxiety, general motor function, or depression-like behaviors (Supplementary Fig. 5b-f, h, i, k, I). We found that the *Kmt2c*^+/fs^ mice showed enhancement of PPI, but this might be explained by decreased acoustic startle responses in the mutants (Supplementary Fig. 5g).

Taken together, *Kmt2c*^+/fs^ mice showed lower sociality in the three-chamber social interaction test, lower working memory in the Y-maze test, inflexibility in the probe test of Barnes maze and the serial reversal test of IntelliCage, and increased acoustic startle response. These phenotypes recapitulate the core symptoms of ASD, impaired social communication and interaction and restricted or repetitive behaviors, as well as auditory hypersensitivity and cognitive impairments frequently observed in the patients. Thus, we considered that *Kmt2c*^+/fs^ mice have a sufficient level of face validity as an ASD model.

### Enrichment of ASD genetic risks among the genes upregulated, but not downregulated, in *Kmt2c*^+/fs^ mouse forebrain

**Fig. 2:**
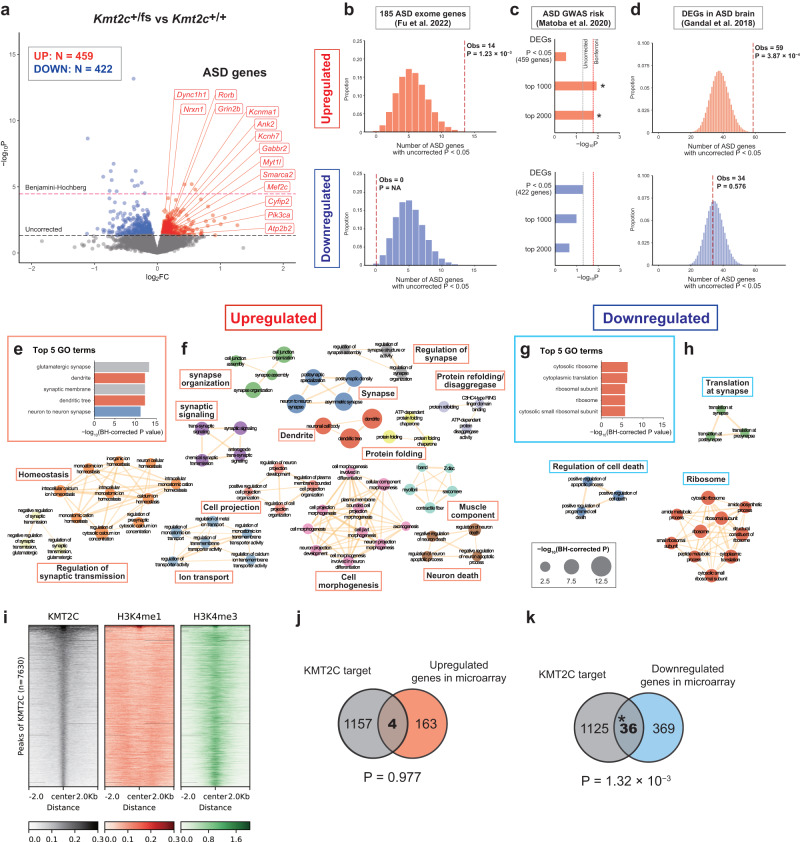
Enrichment of ASD generic risks in genes upregulated in *Kmt2c*^+/fs^ mice as a putative consequence of the indirect effects of *Kmt2c* haploinsufficiency. **a** Volcano plot of differential gene expression analysis by RNA-seq of bulk forebrain samples from adult *Kmt2c*^+/fs^ and *Kmt2c*^+/+^ mice (N = 4 for each genotype, 11 weeks old). The red and blue circles indicate 459 upregulated and 422 downregulated DEGs with uncorrected *P* value < 0.05, respectively. DEGs with uncorrected *P* value < 0.05 that are included in the list of 185 ASD exome genes identified by Fu et al. [10] are shown with gene symbol labels. The red and gray dotted horizontal lines indicate Benjamini-Hochberg (BH) corrected *P* = 0.05 and uncorrected *P* = 0.05, respectively. FC, Fold change. **b** Overlap between the 185 ASD exome genes and upregulated (top) or downregulated (bottom) DEGs with uncorrected P value < 0.05 in *Kmt2c*^+/fs^ mice. The histograms show the hypergeometric distributions of the expected numbers of overlapping genes. The red dotted vertical lines indicate the observed counts of overlapping genes along with the corresponding hypergeometric *P* values. **c** An LDSC analysis testing enrichment of SNP-based ASD heritability in upregulated (top) and downregulated (bottom) DEGs in *Kmt2c*^+/fs^ mice with uncorrected *P* value < 0.05, top 1000 DEGs, and top 2000 DEGs. The summary statistics in an ASD GWAS meta-analysis by Matoba et al. [37] were used. The gray and red dotted vertical lines indicate uncorrected *P* = 0.05 and Bonferroni-corrected *P* = 0.05 (uncorrected *P* × 3 [3 analyses]), respectively. *Bonferroni-corrected *P* < 0.05. **d** Overlap between DEGs in the postmortem human ASD brains identified by Gandal et al. [32] and upregulated (top) or downregulated (bottom) DEGs with uncorrected *P* value < 0.05 in *Kmt2c*^+/fs^ mice. The histograms show the hypergeometric distributions of the expected numbers of overlapping genes. The red dotted vertical lines indicate the observed counts of overlapping genes along with the corresponding hypergeometric *P* values. **e** Top 5 GO terms most significantly enriched in upregulated DEGs with uncorrected *P* value < 0.05 in *Kmt2c*^+/fs^ mice, as identified by a Metascape [40] analysis. The color of the bars corresponds to that of the nodes in (**f**). **f** Network visualization of the GO terms significantly enriched in upregulated DEGs with uncorrected *P* value < 0.05 in *Kmt2c*^+/fs^ mice using Cytoscape [41]. Significant GO terms with BH-corrected *P* < 0.05 are visualized in the network. The sizes of nodes are proportional to the statistical significance ( − log_10_(BH-corrected *P* value)). Edges are drawn when the Kappa coefficient of two nodes is greater than 0.6, and their width is proportional to the Kappa coefficient. Nodes are color-coded by clusters. The networks consisting of less than 3 nodes are not displayed. **g** Top 5 GO terms most significantly enriched in downregulated DEGs with uncorrected *P* value < 0.05 in *Kmt2c*^+/fs^ mice. **h** Network visualization of the GO terms significantly enriched in downregulated DEGs with uncorrected *P* value < 0.05 in *Kmt2c*^+/fs^ mice. **i** Heatmaps of ChIP-seq coverages for KMT2C, H3K4me1, and H3K4me3 in the regions 2 kb upstream and downstream of the center of KMT2C peaks (N = 7630). All heatmaps are sorted in the same order by the mean of the KMT2C signal. The ChIP-seq data for KMT2C was obtained in this study using hippocampal samples (N = 2, 16 weeks old wildtype male mice). This region was selected because we verified the specificity of the KMT2C antibody in hippocampus. The data for H3K4me1 and H3K4me3 were obtained from a previous study of hippocampal samples from 2 − 4 months-old male mice [48]. The KMT2C peaks colocalize with H3K4me3, rather than H3K4me1 signals. **j**, **k** Overlap between KMT2C target genes identified by ChIP-seq and upregulated (**j**) or downregulated (**k**) DEGs in *Kmt2c*^+/fs^ mice. These DEGs were identified by a DNA microarray analysis using hippocampal samples from *Kmt2c*^+/+^ and *Kmt2c*^+/fs^ male mice (N = 5 each, 16 weeks old). The significance of the observed numbers of overlapping genes was calculated by hypergeometric tests.

To identify the molecular alterations that may underlie the observed ASD-like phenotypes in *Kmt2c*^+/fs^ mice, we performed RNA-seq of bulk adult (11 weeks old) forebrain samples mainly including the prefrontal cortex, whose important roles in neuropsychiatric disorders have been consistently demonstrated [32, 47]. By analyzing four mice per genotype, we identified 3 upregulated and 14 downregulated differentially expressed genes (DEGs) at Benjamini-Hochberg-corrected *P* < 0.05, and 459 upregulated and 422 downregulated DEGs at uncorrected *P* value < 0.05 (Fig. 2a and Supplementary Table 2a). Considering that the number of significant DEGs at Benjamini-Hochberg-corrected *P* < 0.05 might be too small to draw biologically meaningful information, we primarily used the lists of 459 upregulated and 422 downregulated DEGs at uncorrected *P* < 0.05 in the subsequent bioinformatics analyses.

First, we examined if these DEGs are enriched for known ASD genetic risks, using results from large-scale human genetics studies of both rare and common variants [10, 37] as well as transcriptomic analysis of human postmortem brains [32]. When we assessed the overlap between the DEGs in *Kmt2c*^+/fs^ mice and the 185 ASD-associated genes (at Benjamini-Hochberg-corrected *P* < 0.05) in a recent large-scale exome-wide rare variant study [10] (hereafter the 185 ASD exome genes), we observed that the upregulated DEGs are significantly enriched for these 185 ASD exome genes (Fig. 2b, *P* = 1.23 × 10^−3^, hypergeometric test). On the other hand, there was no overlap between the downregulated DEGs and the 185 ASD exome genes (Fig. 2b). In a stratified Linkage Disequilibrium SCore regression (LDSC) analysis [38, 39], testing if common single nucleotide polymorphisms (SNPs) in human genes orthologous to the DEGs of *Kmt2c*^+/fs^ mice are enriched for ASD heritability in a recent meta-analysis of genome-wide association studies (GWAS) [37], we did not observe significant enrichment in both upregulated and downregulated DEGs (Fig. 2c and Supplementary Table 2b). Meanwhile, when we performed an LDSC analysis using different thresholds for DEG selection and subjected the top 1000 or 2000 upregulated and downregulated DEGs sorted by *P* value, following the strategy used in a previous study [39] (see Materials and methods for details), we observed significant SNP heritability enrichment in the upregulated DEGs (Fig. 2c, *P* = 0.0116 for top 1000 genes and 0.0157 for top 2000 genes). Again, the downregulated DEGs showed no significant enrichment (Fig. 2c, *P* = 0.102 for top 1000 genes and 0.217 for top 2000 genes). We observed a significant overlap between the upregulated DEGs in *Kmt2c*^+/fs^ mice and the significant DEGs (Benjamini-Hochberg-corrected *P* < 0.05) in human postmortem ASD brains [32] (Fig. 2d, *P* = 3.87 × 10^−4^, hypergeometric test), whereas there was no significant overlap between the downregulated DEGs in the mutant mice and the DEGs in human ASD brains (Fig. 2d, *P* = 0.576, hypergeometric test).

Next, we performed a GO enrichment analysis using Metascape [40] to obtain general insights into which molecular pathways and functions are affected by the *Kmt2c* haploinsufficiency (Fig. 2e-h and Supplementary Table 2c, d). We identified 204 and 19 GO terms significantly enriched at Benjamini-Hochberg-corrected *P* < 0.05 in the upregulated and downregulated DEGs, respectively. In the upregulated DEGs, we found that GO terms for neuronal components, such as synapse and dendrite, were most significantly enriched (Fig. 2e), whereas terms associated with ribosome were strongly enriched in the downregulated DEGs (Fig. 2g). Network visualization of the enriched GO terms showed that functional clusters formed by the terms enriched in the upregulated DEGs include those associated with synapse, ion transport, cell projection and morphogenesis, and other functions (Fig. 2f), and that there are two clusters of terms associated with ribosome and cell death regulation in the downregulated DEGs (Fig. 2h).

Overall, these results indicate that genes upregulated, but not downregulated, in *Kmt2c*^+/fs^ mouse forebrain are enriched for ASD genetic risks and pathways and cellular components assumed to play key roles in the ASD pathogenesis, such as synaptic transmission and projection and morphogenesis of cells including neurons.

### Colocalization of KMT2C with H3K4me3 and downregulated DEGs in the brain

Given the function of KMT2C, it is expected that the observed transcriptomic alterations are mediated by the reduced histone-modifying activity in *Kmt2c*^+/fs^ mice. To better understand the mechanistic basis, we then performed chromatin immunoprecipitation followed by sequencing (ChIP-seq) to comprehensively map the genomic regions bound to KMT2C in the brain of adult wildtype mice. We analyzed two biological replicates and detected 7630 KMT2C peaks (Supplementary Table 4a, see Materials and methods for details). We then assessed the colocalization of the KMT2C peaks with H3K4me1 or H3K4me3 ChIP-seq peaks in the mouse hippocampus using the data from a previous study [48]. By visualizing the overlaps using deeptools, we observed that KMT2C peaks colocalize with H3K4me3 peaks rather than H3K4me1 (Fig. 2i). This result was somewhat unexpected because KMT2C is known to mediate mono- and di-methylation of histone H3 and its association with enhancers has been reported [49–51], however, a similar pattern of colocalization was reproduced when we used an independent ChIP-seq data of H3K4me1 and H3K4me3 in cortical samples obtained via ENCODE (https://www.encodeproject.org/) (Supplementary Fig. 6). Therefore, KMT2C may contribute to the regulation of promoters and transcription start sites (TSSs) marked by H3K4me3 rather than enhancers in the adult mouse brain.

Related to this observation, we analyzed the overlap between the genes whose promoter-TSS is bound to KMT2C (KMT2C target genes) and DEGs in the hippocampus of adult *Kmt2c*^+/fs^ mice, which were screened by a DNA microarray analysis (Supplementary Table 4b). While the KMT2C target genes did not significantly overlap with upregulated DEGs (Fig. 2j and Supplementary Table 4c, *P* = 0.977, hypergeometric test), there was significant overlap between the KMT2C targets and downregulated DEGs (Fig. 2k and Supplementary Table 4c, *P* = 1.32 × 10^−3^, hypergeometric test). This result is reasonable because H3K4 methylation that can be mediated by KMT2C is in general considered to induce transcriptional activation [52, 53]. From another view, it was suggested that upregulated DEGs in *Kmt2c*^+/fs^ mice, which are significantly enriched for genes associated with ASD, are the consequence of indirect effects of the *Kmt2c* haploinsufficiency. Speculating the properties of genes that are directly regulated by KMT2C, the GO term most significantly enriched in the 36 KMT2C target genes that were downregulated in the hippocampus was “negative regulation of translation” (Supplementary Table 4d, uncorrected *P* = 4.12 × 10^−3^). While this enrichment was nominal due to the small number of input genes, haploinsufficiency of *Kmt2c* may cause the downregulation of these genes through its direct effects and then induces ASD-associated transcriptomic alteration enriched in the upregulated DEGs.

### Identification of neonatal cell types with pronounced ASD-associated transcriptomic alterations in *Kmt2c*^+/fs^ mice by integrative scRNA-seq analysis

**Fig. 3:**
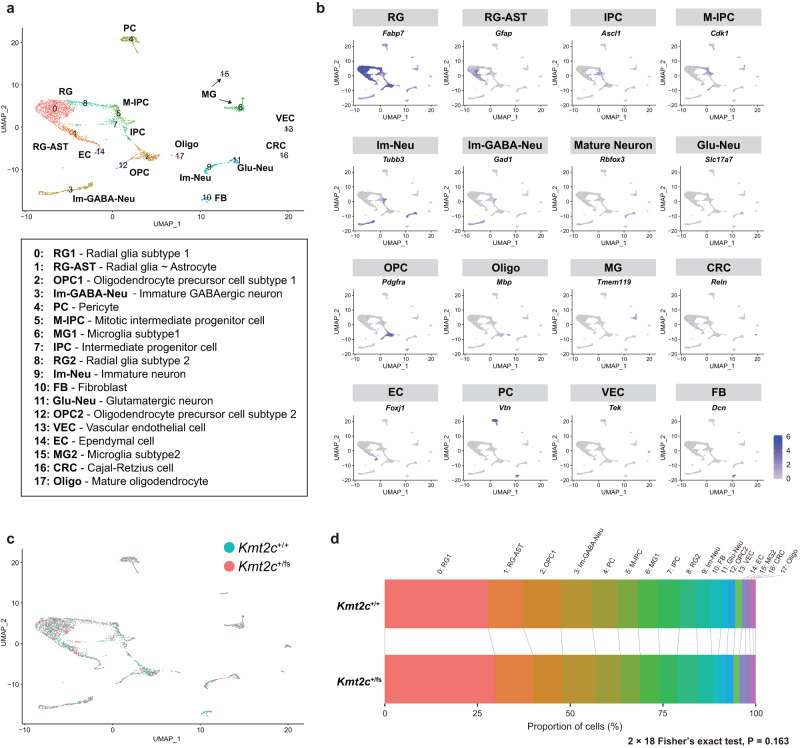
Cell type compositions of *Kmt2c*^+/+^ and *Kmt2c*^+/fs^ mouse neonatal forebrains as revealed by scRNA-seq. **a** UMAP embedding of the scRNA-seq data of P4 neonatal forebrain samples from *Kmt2c*^+/+^ and *Kmt2c*^+/fs^ mice (N = 3 each). We profiled transcriptomes of 4162 cells (*Kmt2c*^+/+^, 2069 cells; *Kmt2c*^+/fs^, 2093 cells) and identified 18 cell clusters as indicated in the box. Cell types were annotated based on the expression patterns of known marker genes as follows. 0: RG1 - Radial glia subtype 1, 1: RG-AST - Radial glia ~ Astrocyte, 2: OPC1 - Oligodendrocyte precursor cell subtype 1, 3: Im-GABA-Neu - Immature GABAergic neuron, 4: PC - Pericyte, 5: M-IPC - Mitotic intermediate progenitor cell, 6: MG1 - Microglia subtype 1, 7: IPC - Intermediate progenitor cell, 8: RG2 - Radial glia subtype 2, 9: Im-Neu - Immature neuron, 10: FB - Fibroblast, 11: Glu-Neu - Glutamatergic neuron, 12: OPC2 - Oligodendrocyte precursor cell subtype 2, 13: VEC - Vascular endothelial cell, 14: EC - Ependymal cell, 15: MG2 - Microglia subtype 2, 16: CRC - Cajal-Retzius cell, 17: Oligo - Mature oligodendrocyte. **b** Expression patterns of representative marker genes. **c** UMAP embedding colored by the genotype. *Kmt2c*^+/+^, blue; *Kmt2c*^+/fs^, red. **d** Proportional compositions of 18 cell type clusters in *Kmt2c*^+/+^ and *Kmt2c*^+/fs^ mouse neonatal forebrains. The difference between *Kmt2c*^+/+^ and *Kmt2c*^+/fs^ mice was assessed by 2 × 18 Fisher’s exact test.

**Fig. 4:**
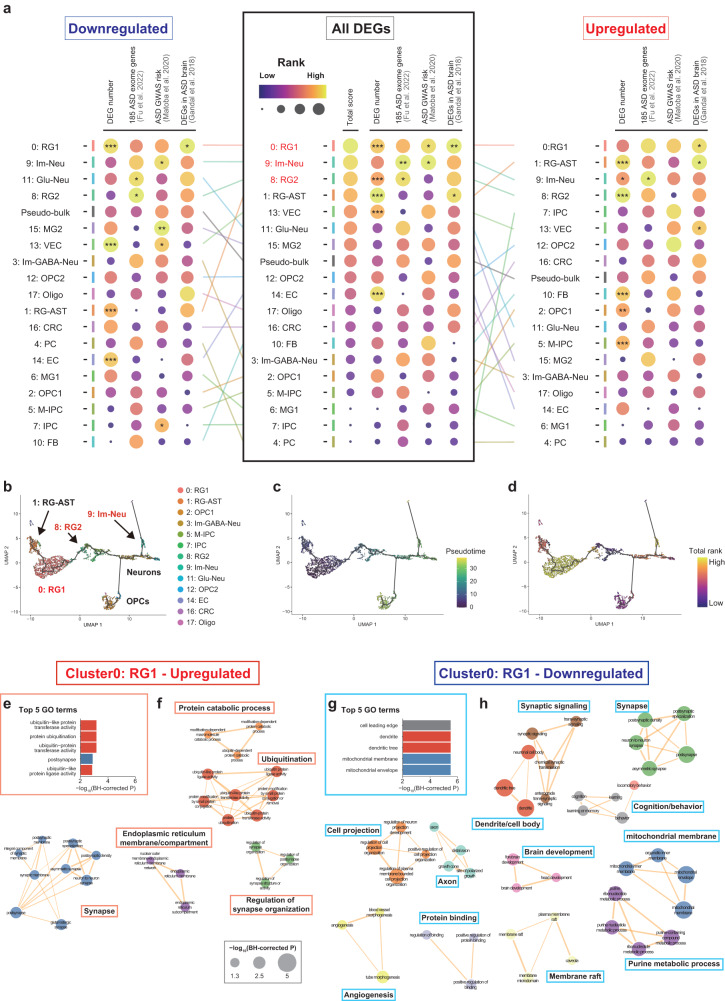
Characterization of neonatal cell types with ASD-associated transcriptomic alterations in *Kmt2c*^+/fs^ mice. **a** Identification of cell type clusters with ASD-associated transcriptomic alterations in *Kmt2c*^+/fs^ mice. As described in the main text and Materials and methods, we identified and analyzed DEGs (uncorrected *P* value < 0.05) in each cell cluster and ranked the clusters by 1) positive deviation from the expected DEG numbers, 2) overlap with the 185 ASD exome genes, 3) enrichment of SNP heritability in ASD GWAS, and 4) overlap with the DEGs in the ASD postmortem brains. Then we prioritized the cell clusters with pronounced ASD-associated transcriptomic alterations based on the sum of the ranks in these four analyses (“Total scores” in the figure). The results of the analysis for downregulated (left), all (middle), and upregulated (right) DEGs are shown. The lines between the result for all DEGs and downregulated or upregulated DEGs represent changes in the ranking, which are colored as in the cell type clusters in Fig. 3. The colors and the sizes of the circles correspond to their ranks, as shown in the lower right. ***significant after Bonferroni correction (uncorrected *P* value < 0.05/228 [19 clusters × 4 analyses × 3 groups of DEGs]); **significant after Benjamini-Hochberg (BH) correction; *nominally significant (uncorrected *P* value < 0.05). The clusters showing the same total scores were ranked by combining the four *P* values for each cluster by Fisher’s method. **b**–**d** Pseudo-time trajectory analysis of neuronal-, oligodendrocyte- and astrocyte-lineage cell clusters by Monocle3. The UMAP representations colored by cell clusters in Fig. 3 (**b**), pseudo-time (**c**), or the total score in panel (**a**), (**d**) are shown. 8: RG2 was shown to differentiate into 9: Im-Neu and oligodendrocyte-lineage cells. **e**, **g** The top five significantly enriched GO terms in the upregulated (**e**) or downregulated (**g**) DEGs in cluster 0: RG1 identified by Metascape [40]. The colors of the bars correspond to that of the network figures in (**f**, **h**). The full lists of enriched GO terms are shown in Supplementary Tables 5g and 5h. **f**, **h** Network visualization of the GO terms enriched in the upregulated (**f**) or downregulated (**h**) DEGs in cluster 0: RG1 by Cytoscape [41]. Significant GO terms with BH-corrected *P* < 0.05 are visualized in the network. The size of nodes indicates the statistical significance ( − log10(BH-corrected *P* value)). The edge width is proportional to the similarity scores calculated by Kappa-test between two nodes [40], and the threshold of the score is 0.6. The nodes are color-coded by the GO cluster. The networks consisting of three or more nodes are included in the figure.

While the above analyses examined transcriptomic dysregulation in the bulk brain of adult *Kmt2c*^+/fs^ mice, in which we observed ASD-like behavioral changes, further insights can be obtained by investigating an earlier developmental stage of the brain in a cell type-resolved manner, given the developmental nature of ASD. To this end, we performed scRNA-seq of neonatal (postnatal day 4: P4) forebrain samples from *Kmt2c*^+/fs^ mice and their wildtype littermates (Figs. 3 and 4). We selected this stage because *Kmt2c* is most highly expressed at P4 according to the data in the Allen Developing Mouse Brain Atlas (https://developingmouse.brain-map.org/). Among several different approaches for scRNA-seq, we employed Quartz-seq2, which showed the best overall score in a recent benchmark study comparing 13 methods [54]. After the cell sorting, we obtained transcriptomic profiles of a total of 4,162 cells (*Kmt2c*^+/+^, 2069 cells; *Kmt2c*^+/fs^, 2093 cells) passing the quality control (Supplementary Fig. 2, see Materials and methods for details). We performed unsupervised clustering and data visualization by uniform manifold approximation and projection (UMAP) and identified 18 cell clusters, which were labeled by expression patterns of known cell type markers (Fig. 3a, b and Supplementary Fig. 7). When we assessed the proportional compositions of cell types based on these 18 clusters, there was no gross difference between *Kmt2c*^+/fs^ and wildtype mice (Fig. 3c, d, *P* = 0.163, 2 × 18 Fisher’s exact test). An analysis of individual clusters showed nominal decreases of 2: Oligodendrocyte precursor cell subtype 1 (OPC1) and 10: Fibroblast (FB) in *Kmt2c*^+/fs^ mice (uncorrected *P* = 0.0359 for 2: OPC1 and uncorrected *P* = 0.0171 for 10: FB, 2 × 2 Fisher’s exact test), while these are not significant after multiple testing correction.

To identify the cell types that would contribute more to the pathological changes in *Kmt2c*^+/fs^ mice, we performed the following analyses that utilize an integrative approach. First, we identified DEGs (uncorrected *P* value < 0.05) in each cluster using MAST (Model-based Analysis of Single-cell Transcriptomics) [55]. Whereas the number of DEGs of each cell cluster showed a significantly positive correlation with the number of cells (Supplementary Fig. 8, *P* = 1.50 × 10^−16^, Pearson’s correlation coefficient r = 0.925), we observed a portion of the observed number of DEGs deviated from the regression line. After that, we analyzed the DEGs in each cell cluster and ranked the clusters based on 1) positive deviation from the expected number of DEGs, 2) overlap with the 185 ASD exome genes [10], 3) enrichment of ASD SNP heritability in GWAS [37], and 4) overlap with the DEGs in the ASD postmortem brains [32]. We then prioritized the cell clusters with pronounced ASD-associated transcriptomic alterations according to the sum of the ranks in these four analyses (see Materials and methods for details). These analyses were performed for all, upregulated, and downregulated DEGs (Fig. 4a, Supplementary Tables 3 and 5a-f). In the analysis of all (both upregulated and downregulated) DEGs, cluster 0: radial glia subtype 1 (RG1) exhibited the highest total score (Fig. 4a, middle), followed by cluster 9: immature neuron (Im-Neu) and cluster 8: RG2. When we analyzed upregulated and downregulated DEGs separately, 0: RG1 was commonly ranked at the top, indicating pervasive ASD-associated transcriptomic alterations in this cell cluster regardless of the direction of expression changes. Overall, the other clusters also did not exhibit ASD-associated transcriptomic alterations specific in upregulated DEGs, unlike the observation in the bulk brain of adult *Kmt2c*^+/fs^ mice. When we evaluated the correlation between the cell cluster ranks in the upregulated and downregulated DEGs, there was no significant correlation with *P* = 0.0986 (Fig. 4a, left and right, Spearman’s correlation coefficient ρ = 0.391).

To characterize cell clusters in the context of cell differentiation, we then performed a pseudo-time trajectory analysis of neuronal-, oligodendrocyte- and astrocyte-lineage cells [56] using Monocle3 [56, 57]. We found that the 0: RG1 cluster with the most pronounced ASD-associated transcriptomic alteration in the analysis of all DEGs (Fig. 4a, middle) represents cells in the most undifferentiated state (Fig. 4b-d). The 8: RG2 cluster and the 1: Radial glia-Astrocyte (RG-AST) cluster, which respectively showed the third and fourth most pronounced ASD-associated alteration, were identified as cells differentiated from the 0: RG1 cluster (Fig. 4b-d). Intermediate progenitor cell clusters (e.g., 5: M-IPC, 7: IPC) were mapped as originating from the 8: RG2 cluster, and differentiation of these cells into the neuronal cell lineage, including the 9: Im-Neu with the second most pronounced ASD-associated alteration and oligodendrocyte precursor cells was delineated (Fig. 4b-d).

When we characterized the DEGs in cluster 0: RG1 by a GO enrichment analysis, we found that synaptic terms are commonly enriched among upregulated and downregulated DEGs (Fig. 4e-h, Supplementary Tables 5g and h). On the other hand, terms associated with ubiquitination were shown to be strongly enriched in upregulated DEGs, and enrichment of those associated with dendrite and mitochondrial membrane was prominent in downregulated DEGs (Fig. 4e-h, Supplementary Tables 5g, h). For the clusters 9: Im-Neu and 8: RG2, which showed the second and the third most pronounced ASD-associated transcriptomic alterations, respectively (Fig. 4a, middle), terms such as “glutamatergic synapse”, “synaptic membrane”, “cellular component morphogenesis”, and “mitochondrial envelope” were significantly enriched in upregulated DEGs in the cluster 9: Im-Neu, while the significant terms in upregulated DEGs in the cluster 8: RG2 include “DNA metabolic process” and “DNA replication” (Supplementary Fig. 9 and Supplementary Table 5i-l). In contrast, we did not observe a formation of networks of significant GO terms in downregulated DEGs in these clusters (Supplementary Fig. 9 and Supplementary Table 5i–l).

Overall, we found that transcriptomic alteration of genes associated with ASD risk and ASD-related biological components, such as synapses, was prominent in undifferentiated radial glial cells and those differentiating into the neuronal lineage in the neonatal brain of *Kmt2c*^+/fs^ mice. This may constitute the basis of the observations in the adult mutant mice. On the other hand, we did not observe a gross change of cellular composition in the neonatal *Kmt2c*^+/fs^ mice brain, suggesting that transcriptomic dysregulation in undifferentiated brain cells (e.g., 0: RG1 and 8: RG2) due to *Kmt2c* haploinsufficiency does not drastically alter cell fates.

### An inhibitor of histone demethylase LSD1 rescues social deficits and transcriptomic dysregulation in *Kmt2c*^+/fs^ mice

Our RNA-seq analyses of adult and neonatal brains characterize transcriptomic dysregulation due to the haploinsufficiency of *Kmt2c*. Meanwhile, we did not observe a major change in cellular composition in the neonatal brain of the mutant mice. This may suggest that there remained the possibility that modulation of transcriptional regulation in adult *Kmt2c*^+/fs^ mice could improve their phenotypic outcomes. In this context, we explored the therapeutic effects of pharmaceutical intervention targeting histone modification.

**Fig. 5:**
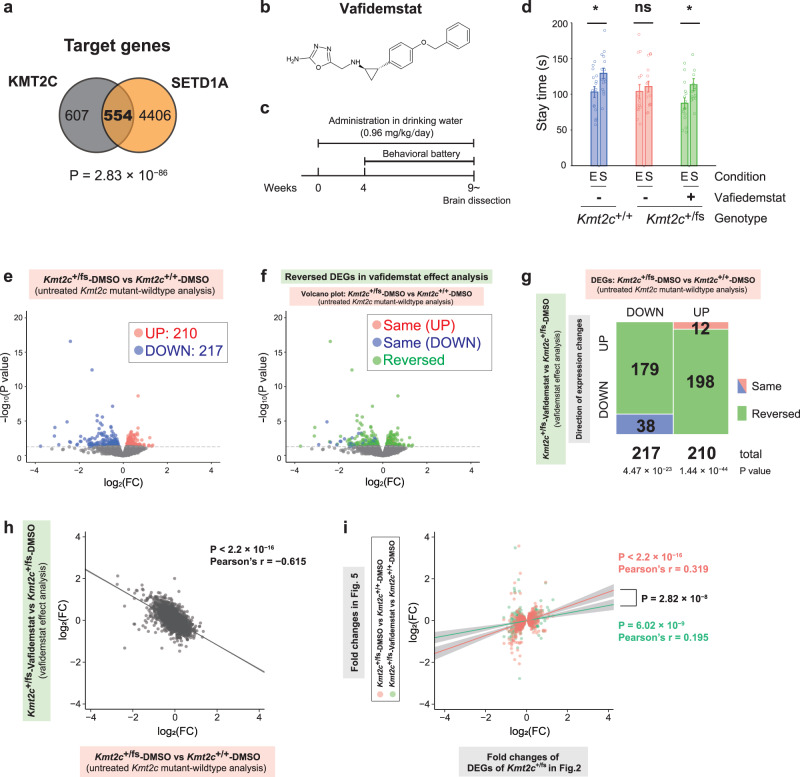
Rescue of social deficits and transcriptomic alterations in *Kmt2c*^+/fs^ mice by an LSD1 inhibitor, vafidemstat. **a** Overlap of the target genes of KMT2C and SETD1A. There is a significant overlap between the protein-coding genes whose promotor-TSS regions are bound by KMT2C and those bound by SETD1A. A hypergeometric test was used for statistical comparison. **b** The structural formula of vafidemstat drawn by ChemSketch. **c** Schematic representation of the experimental schedule. Administration of vafidemstat was started 4 weeks before the behavioral tests consisting of the open field, Y-maze, three-chamber social interaction, and prepulse inhibition tests. The brain samples were dissected after the behavioral test battery. Vafidemstat was administered via drinking water with 0.96 mg/kg/day. **d** Effect of vafidemstat on sociality in the three-chamber social interaction test. Though *Kmt2c*^+/fs^ mice treated with vehicle (DMSO) did not exhibit significantly longer stay time near the stranger mice (*P* = 0.586, red bars), DMSO-treated *Kmt2c*^+/+^ and vafidemstat-treated *Kmt2c*^+/fs^ mice showed significant difference (*P* = 0.0190 for DMSO-treated *Kmt2c*^+/+^ mice [blue bars] and *P* = 0.0314 for vafidemstat-treated *Kmt2c*^+/fs^ mice [green bars]). (N = 15 in each condition). Welch’s t-test was used for statistical comparison between the stay time in the empty (E) and stranger (S) chambers. *P < 0.05. The data are presented as mean ± standard error of the mean (SEM). **e**, **f** Volcano plot of differential gene expression analysis comparing DMSO-treated *Kmt2c*^+/fs^ and DMSO-treated *Kmt2c*^+/+^ mice (“untreated *Kmt2c* mutant-wildtype analysis” in the main text). A total of 210 upregulated and 217 downregulated DEGs (uncorrected *P* < 0.05) were identified by RNA-seq of bulk forebrain samples (*Kmt2c*^+/+^, N = 3; *Kmt2c*^+/fs^, N = 2, 22 weeks old). The red and blue circles in (**e**) represent the upregulated and downregulated DEGs, respectively. In (**f**), the green circles indicate the DEGs in untreated *Kmt2c* mutant-wildtype analysis whose expression levels were changed in the opposite direction (reversed), i.e., normalized toward the status of wildtype mice, in a comparison between vafidemstat-treated *Kmt2c*^+/fs^ and DMSO-treated *Kmt2c*^+/fs^ mice (“vafidemstat effect analysis“ in the main text). The other DEGs are indicated by the red and blue circles as in (**e**). In (**e**, **f**) the gray dotted horizontal lines indicate uncorrected *P* < 0.05 and the gray circles indicate non-DEGs. **g** Mosaic plot of the 210 upregulated and 217 downregulated DEGs in untreated *Kmt2c* mutant-wildtype analysis according to the direction of their expression changes in the vafidemstat effect analysis. As shown in the green areas of the figure, 198 of the 210 upregulated DEGs were decreased (*P* = 1.44 × 10^−44^) and 179 of the 217 downregulated DEGs were increased (*P* = 4.47 × 10^−23^) in the vafidemstat effect analysis. Binomial tests were used for statistical comparison. **h** Scatter plot of the fold changes of each gene in the untreated *Kmt2c* mutant-wildtype analysis (the X-axis) and the fold changes in the vafidemstat effect analysis (the Y-axis). Pearson’s correlation coefficient (r) and the corresponding *P* value are shown in the upper right of the panel. The black line and the (very narrow) gray area surrounding the line represent the regression line and its 95% confidence interval, respectively. **i** Scatter plot of the fold changes of significant DEGs in *Kmt2c*^+/fs^ mice identified in Fig. 2 (the X-axis) and the fold changes of these genes observed in the untreated *Kmt2c* mutant-wildtype analysis (red circles) or those observed in a comparison between vafidemstat-treated *Kmt2c*^+/fs^ and DMSO-treated *Kmt2c*^+/+^ mice (i.e., an analysis of treated mutant and untreated wildtype mice, green circles) (the Y-axis). The red and green lines indicate the corresponding regression lines. The gray areas surrounding the regression line represent the 95% confidence intervals. Pearson’s correlation coefficients (r) and the corresponding *P* values for each analysis, indicated by red or green, are shown. We observed that the correlation coefficient observed for the fold changes in the untreated *Kmt2c* mutant-wildtype analysis (0.319, red) is significantly different from that observed for the fold changes in the analysis of treated mutant and untreated wildtype mice (0.195, green) (*P* = 2.82 × 10^−8^, Steiger’s method [42]).

In identifying potential therapeutics, we noticed that the KMT2C target genes detected by ChIP-seq of the adult brain (Fig. 2i-k and Supplementary Table 4a) strikingly overlap with the targets of SETD1A [13] (Supplementary Table 6), another histone methyltransferase catalytic unit known as an established risk gene for schizophrenia and other neurodevelopmental disorders [12, 14, 15], in the brain (Fig. 5a, *P* = 2.83 × 10^−86^, hypergeometric test). In a recent study of the *Setd1a* haploinsufficient mice, it was demonstrated that inhibitors of lysine-specific demethylase LSD1 commonly rescue the alterations of behaviors and neuronal morphology in *Setd1a* heterozygous mutants [13]. Based on these, we speculated that LSD1 inhibition might also be therapeutic in *Kmt2c*^+/fs^ mice, and tested the effect of a brain-penetrant LSD1 inhibitor, vafidemstat [22] (also known as ORY-2001) (Fig. 5b). Unlike Tranylcypromine and ORY-1001 whose effects were evaluated by intraperitoneal injection in a previous study [13], vafidemstat can be administrated via drinking water. We treated *Kmt2c*^+/+^ and *Kmt2c*^+/fs^ mice with vafidemstat at the same doses as previously reported [22] for 4 weeks prior to the behavioral test battery consisting of open field, Y-maze, three-chamber social interaction, and PPI tests (Fig. 5c).

We found that *Kmt2c*^+/fs^ mice administered with vafidemstat and wildtype *Kmt2c*^+/+^ mice spent significantly longer times in the chamber with a stranger mouse (Fig. 5d, *P* = 0.0305 for *Kmt2c*^+/+^ mice and *P* = 0.0314 for *Kmt2c*^+/fs^ mice administered with vafidemstat, Welch’s t-test), whereas no significant difference was observed in *Kmt2c*^+/fs^ mice treated with a vehicle (dimethyl-sulfoxide [DMSO]) (*P* = 0.586). In the other tests, we did not observe significant amelioration of the behavioral abnormalities (Supplementary Fig. 10), suggesting that vafidemstat is effective for specific, but not all, behavioral phenotypes.

To elucidate the molecular mechanisms underlying the rescue effects of vafidemstat on ASD-like behaviors, we conducted bulk RNA-seq using the forebrain samples from the untreated and treated mice after the behavioral tests. When we performed a comparison between DMSO-treated *Kmt2c*^+/fs^ and DMSO-treated *Kmt2c*^+/+^ mice (hereafter “untreated *Kmt2c* mutant-wildtype analysis”), we identified 210 upregulated and 217 downregulated DEGs at uncorrected *P* value < 0.05 (Fig. 5e and Supplementary Table 7a). We subsequently analyzed vafidemstat-treated *Kmt2c*^+/fs^ and DMSO-treated *Kmt2c*^+/fs^ mice to characterize transcriptomic changes resulting from the administration of vafidemstat in the mutants (hereafter “vafidemstat effect analysis”, Supplementary Table 7b). Notably, we found that the expression levels of 198 of the 210 upregulated DEGs in the untreated *Kmt2c* mutant-wildtype analysis (94.3%) were decreased (reversed) in the vafidemstat effect analysis, an observation highly unlikely to have occurred by chance (Fig. 5f, g and Supplementary Table 7c, *P* = 1.44 × 10^−44^, binomial test). Similarly, 179 of the 217 downregulated DEGs in the untreated *Kmt2c* mutant-wildtype analysis (82.5%) were increased in the vafidemstat effect analysis (Fig. 5f, g and Supplementary Table 7c, *P* = 4.47 × 10^−23^, binomial test). In addition, we observed a significant negative correlation between the fold changes in the untreated *Kmt2c* mutant-wildtype analysis (the X-axis in Fig. 5h) and the fold changes in the vafidemstat effect analysis (the Y-axis in Fig. 5h) (*P* < 2.2 × 10^−16^, Pearson’s correlation coefficient r = −0.615), indicating that vafidemstat exerts an overall restoring effect on the transcriptomic alterations in *Kmt2c*^+/fs^ mice, which is not restricted to significant DEGs. To further verify the effects of vafidemstat on the transcriptome, we analyzed how the overall profile of DEGs observed in the adult forebrain of *Kmt2c*^+/fs^ mice, shown in Fig. 2a (uncorrected *P* < 0.05, 459 upregulated and 422 downregulated genes), can be altered by the administration of this drug. We first found that the fold changes of the DEGs identified in Fig. 2 were highly significantly correlated with the fold changes observed in the untreated *Kmt2c* mutant-wildtype analysis (the red dots and regression line in Fig. 5i, *P* < 2.2 × 10^−16^, Pearson’s r = 0.319), which is independent of the analysis in Fig. 2a and was performed after the behavioral analysis. Next, we assessed the correlation between the fold changes of the DEGs in Fig. 2a and the fold changes observed in a comparison between vafidemstat-treated *Kmt2c*^+/fs^ and DMSO-treated *Kmt2c*^+/+^ mice (i.e., an analysis of treated mutant and untreated wildtype mice) (Supplementary Table 7d). In this analysis, we still observed a significant positive correlation (the green dots and regression line in Fig. 5i, *P* = 6.02 × 10^−9^, Pearson’s r = 0.195); however, the correlation coefficient (r) was significantly lower than that obtained from the above-described analysis of fold changes in Fig. 2a and those in the untreated *Kmt2c* mutant-wildtype analysis (Fig. 5i, 0.319 v.s. 0.195, *P* = 2.87 × 10^−8^, calculated by a test for comparing dependent two correlations proposed by Steiger [42]). These observations collectively indicate that the transcriptomic alterations *Kmt2c*^+/fs^ mice were overall normalized toward the state in the wildtype mice by the vafidemstat treatment, and this can be the molecular mechanism underlying its alleviating effect on sociality deficits.

## Discussion

We, in the present study, investigated the consequence of the haploinsufficiency of a histone methyltransferase gene *KMT2C*, whose heterozygous LOF variants have been identified as a robust causal risk factor for ASD and NDD in large-scale human genetics studies, by creating *Kmt2c*^+/fs^ mice. Overall, the mutant mice manifested multiple ASD-related phenotypes in their gross brain development and behaviors, such as megalencephaly, social deficits, lack of flexibility, cognitive impairment, and auditory hypersensitivity (Fig. 1), supporting the face validity of *Kmt2c*^+/fs^ mice as an ASD model. Therefore, the product of the current study contributes to the expansion of the repertoire of high-validity ASD mouse models, and in particular, it can be a useful tool for elucidating the mechanistic basis of how dysregulation of chromatin modifications, one of the major biological processes involved in the ASD etiology, lead to its pathogenesis.

In this context, we performed a series of transcriptomic and epigenomic profiling of the brain of *Kmt2c*^+/fs^ mice (Figs. 2–4). In our analysis of bulk adult forebrain, we observed that ASD genetic risks, DEGs in human ASD postmortem brains, and genes involved in ASD-related biological components (e.g., synapses) are particularly enriched in upregulated DEGs in *Kmt2c*^+/fs^ mice. This was somewhat unexpected as KMT2C mediates H3K4 methylation, which is generally thought to activate gene expression [52, 53], and thereby *Kmt2c* haploinsufficiency is expected to cause the downregulation of target genes. Subsequent ChIP-seq analysis confirmed that KMT2C target genes significantly overlap with downregulated DEGs but not with upregulated ones, implying that *Kmt2c* haploinsufficiency leads to ASD-associated transcriptomic alteration through its indirect effects on gene expression (Fig. 2). This would be in line with the results observed in a study of *CHD8*, another robust ASD gene involved in chromatin modification, reporting the importance of its indirect effects [58].

In the scRNA-seq analysis of the neonatal forebrain of *Kmt2c*^+/fs^ mice, we employed an integrative approach utilizing the findings from ASD exome sequencing, genome-wide association, and human postmortem brain studies. By characterizing DEGs in each cell cluster, we found that ASD-associated transcriptomic alterations are not specifically pronounced in upregulated DEGs, and are overall most prominent in the undifferentiated radial glia (RG) clusters (Fig. 4). Abnormalities in RG have been reported in multiple genetic and environmental models of ASD, including mice with mutations in *Cntnap2*, *Shank3*, *Tsc2*, *or Foxp1* and those exposed to valproic acid or maternal immune activation [59–62]. Therefore, it would be reasonable to assume that the prominent ASD-associated transcriptomic alteration in RG underlies the abnormalities observed in *Kmt2c*^+/fs^ mice. On the other hand, an analysis based on the results of a large-scale human genetics study [10], which may provide more unbiased information, reported that among various cell types in the human fetal brain, expression of robust ASD risk genes is enriched in the neuronal lineages, especially in excitatory neurons, than in glial cells including RG. Considering this, RG would not be the fetal or neonatal cell type most affected in the general ASD population but rather may play a critical role in specific subtypes of ASD caused by variants in specific genes or environmental insults.

In the last part of this study, we tested the effect of a brain-penetrant LSD1 inhibitor, vafidemstat. We found that vafidemstat ameliorates social deficits in *Kmt2c*^+/fs^ mice, along with a remarkable rescuing effect on transcriptomic alterations in the mutant brain (Fig. 5). This indicates that vafidemstat is a legitimate drug for *Kmt2c*^+/fs^ mice that can help restore the normal transcriptomic state. On the other hand, we did not observe significant therapeutic effects of vafidemstat on other behavioral abnormalities. Given this, transcriptomic restoration in adult mice may not be sufficient to improve all phenotypes. Nevertheless, LSD1 inhibitors have been shown to be effective in other model of neuropsychiatric disorder, as mentioned above, and also human clinical trials are underway. According to the information provided by the developer of vafidemstat (https://www.oryzon.com/en/programs/vafidemstat), there are several clinical trials testing its safety and efficacy. Of these, a Phase IIa clinical trial to examine the effect of vafidemstat on agitation-aggression in patients with several neuropsychiatric disorders including ASD has been performed. This and other studies will more clearly illustrate the potential of vafidemstat as a new class of psychotropic drug.

In summary, here we generated a new mouse model of ASD satisfying both etiological and face validities, which are supported by the causal association of *KMT2C* haploinsufficiency with ASD and multiple autistic-like phenotypes in *Kmt2c*^+/fs^ mice, respectively. Comprehensive analyses of brain gene expression profiles across multiple developmental periods have revealed characteristic transcriptomic dysregulation associated with known ASD genetic risks and ASD-related molecular pathways in the mutants. Our exploration of therapeutic agents based on the known function of KMT2C demonstrated that an LSD1 inhibitor, which is effective in a mouse model of psychiatric disorder with deficiency of another H3K4 methyltransferase gene [13], restores a specific domain of behavioral phenotype as well as transcriptomic alterations in *Kmt2c*^+/fs^ mice. This indicates that histone-modifying drugs would be effective not only in patients with *KMT2C* haploinsufficiency, which represents a tiny proportion of individuals diagnosed with ASD, but also in a broader patient population with specific types of transcriptomic and epigenomic dysregulation. Although further basic and clinical investigations are mandatory, these findings may offer promise in drug discovery for neuropsychiatric disorders, where the development of innovative medication has been largely unsuccessful for decades [63, 64].

## Supplementary information

Supplementary Figures

Supplementary Method

Supplementary Table 1

Supplementary Table 2

Supplementary Table 3

Supplementary Table 4

Supplementary Table 5

Supplementary Table 6

Supplementary Table 7

## Data Availability

Raw data of Figs. 2–5 are available through CBS Data Sharing Platform (10.60178/cbs.20240214-001) or upon request.

## Code availability

Original codes used in this study are available upon request.
