# Supplementary material for: Transcriptomic dysregulation and autistic-like behaviors in Kmt2c haploinsufficient mice rescued by an LSD1 inhibitor
Source: Mol Psychiatry. 2024 Mar 26;29(9):2888–904. doi: 10.1038/s41380-024-02479-8 (PMC11420081; doi:10.1038/s41380-024-02479-8)
Supplement: Supplementary file 1 — Supplementary Figures [file 41380_2024_2479_MOESM1_ESM.docx]

**Supplementary Figures**

**Transcriptomic dysregulation and autistic-like behaviors in *Kmt2c* haploinsufficient mice rescued by an LSD1 inhibitor**

Takumi Nakamura^1, 2, 3, 4^, Toru Yoshihara^5^, Chiharu Tanegashima^6^, Mitsutaka Kadota^6^, Yuki Kobayashi^7^, Kurara Honda^1^, Mizuho Ishiwata^2, 3^, Junko Ueda^1, 3^, Tomonori Hara^1, 8^, Moe Nakanishi^9, 10^, Toru Takumi^9, 11^, Shigeyoshi Itohara^7^, Shigehiro Kuraku^6, 12^, Masahide Asano^5^, Takaoki Kasahara^3, 13^, Kazuo Nakajima^2, 3, 14^, Takashi Tsuboi^4^, Atsushi Takata^1, 15, *^ & Tadafumi Kato^2, 3 *^

*1: Laboratory for Molecular Pathology of Psychiatric Disorders, RIKEN Center for Brain Science, Saitama, Japan.*

*2: Department of Psychiatry and Behavioral Science, Juntendo University Graduate School of Medicine, Tokyo, Japan.*

*3: Laboratory for Molecular Dynamics of Mental Disorders, RIKEN Center for Brain Science, Saitama, Japan.*

*4: Department of Life Sciences, Graduate School of Arts and Sciences, The University of Tokyo, Tokyo, Japan*

*5: Institute of Laboratory Animals, Kyoto University Graduate School of Medicine, Kyoto, Japan*

*6: Laboratory for Phyloinformatics, RIKEN Center for Biosystems Dynamics Research, Hyogo, Japan*

*7: Laboratory for Behavioral Genetics, RIKEN Center for Brain Science, Saitama, Japan*

*8: Department of Organ Anatomy, Tohoku University Graduate School of Medicine, Miyagi, Japan*

*9: Laboratory for Mental Biology, RIKEN Center for Brain Science, Saitama, Japan*

*10: Laboratory for Molecular Mechanism of Brain Development, RIKEN Center for Brain Science, Saitama, Japan*

*11: Department of Physiology and Cell Biology, Kobe University School of Medicine, Hyogo, Japan*

*12: Molecular Life History Laboratory, Department of Genomics and Evolutionary Biology, National Institute of Genetics, Shizuoka, Japan*

*13: Institute of Biology and Environmental Sciences, Carl von Ossietzky University of Oldenburg, Oldenburg, Germany*

*14: Department of Physiology, Teikyo University School of Medicine, Tokyo, Japan*

*15: Research Institute for Diseases of Old Age, Juntendo University Graduate School of Medicine, Tokyo, Japan*

***Correspondence should be addressed to:**

Atsushi Takata, M.D., Ph.D.

Laboratory for Molecular Pathology of Psychiatric Disorders, RIKEN Center for Brain Science, 2-1 Hirosawa, Wako, Saitama, 351-0198, Japan

Phone & Fax: +81-48-467-9703

Email: atsushi.takata@riken.jp

Tadafumi Kato, M.D., Ph.D.

Department of Psychiatry and Behavioral Science, Juntendo University Graduate School of Medicine, 2-1-1 Hongo, Bunkyo-ku, Tokyo, 113-8421, Japan

Phone & Fax: +81-3-5802-1071

Email: [tadafumi.kato@juntendo.ac.jp](mailto:tadafumi.kato@juntendo.ac.jp)

**

**

**Supplementary Fig. 1: The *Kmt2c*^+/fs^ mice showed normality in spatial learning, impulsivity, and attention in IntelliCage analyses.**

**a** Place preference test. The mice could drink water in one of four corners in 21:00-24:00 local time (correct corner) by a nose poke. There was no significance in the rate of visits to the correct corners. N=7 per group. Interaction; P = 0.265, Time; P = 6.94 × 10^−14^, Genotype; P = 0.849. A two-way repeated-measures ANOVA test was used for the statistical comparison. Data are represented as mean ± standard error mean (SEM). **b** Place preference reversal test. In this test, the mice could drink the opposite corner of the correct corner in the place preference test. The *Kmt2c*^+/fs^ mice did not show any significance in the rate to visit the drinking corners. N = 7. Interaction; P = 0.495, Time; P = 2.17 × 10^−16^, Genotype; P = 0.18. A two-way repeated-measures ANOVA test was used for the statistical comparison. Data are represented as mean ± standard error mean (SEM). **c** Impulsivity test. The mice could drink water if they performed a nose poke more than 2 sec after entering the experimental chambers. “Premature” means the mice did an incorrect nose poke. “Omission” means that the mice did not perform a nose poke. N = 7. Interaction; P = 0.0342, Pattern; P < 2 × 10^−16^, Genotype; P > 0.99. A two-way ANOVA test was used for the statistical comparison. Data are represented as mean ± standard error mean (SEM). **d** Attention test. Two gates closing the bottle exist and LED lights are put above the gate in the experimental chamber. In the attention test, One of the two lights above the gates (correct gate) was turned on for 0.3, 0.5, and 1.0 sec, randomly, 4 sec after entering the chamber. The mice could drink water if they perform a nose poke to the correct gate. N = 7. Interaction; P = 0.984, Stimulation; P = 2.04 × 10^−4^, Genotype; P = 0.39. A two-way ANOVA test was used for the statistical comparison. Data are represented as mean ± standard error mean (SEM). **e** Place avoidance test. 2 days before the test, the mice were exposed to air-puff in one of the four corners for 24 hours (training). The mice were transferred to the home cage after the training. The rate of visits to air-puff corners was evaluated in the test session. N = 6. Interaction; P = 0.558, Time; P = 1.13 × 10^−6^, Genotype; P = 0.894. A two-way ANOVA test was used for the statistical comparison. Data are represented as mean ± standard error mean (SEM). **f** Delay discounting test. One of the two bottles contained 0.5% saccharin water in this test. The opening of the gate for saccharin water was delayed compared to the gate for normal water. The delay time was increased by 1 sec every 24 hours. The rate to drink saccharin water was examined every day. N = 6. Interaction; P = 0.638, Time; P = 7.22 × 10^−16^, Genotype; P = 0.282. A two-way repeated-measures ANOVA test was used for the statistical comparison. Data are represented as mean ± standard error mean (SEM).

**

**

**Supplementary Fig. 2: Single cell sorting and quality check of Quartz-seq2 data**

**a** Single-cell sorting in Quartz-seq2. After filtering by the singlet gate, living cells (7-AAD negative) were sorted onto 384 plates. **b** Representative images of cells after the single cell sorting. Scale bar, 50 μm. **c** Scatter plot of gene counts and the mitochondrial ratio of all sequenced cells. The dots are color-coded by the plate ID. Cells with the following conditions were filtered out as outliers; 1) gene count per cell is less than 2,500 or more than 10,000, 2) the proportion of mitochondrial UMI (mitochondrial ratio per cell) is greater than or equal to 12%. After the filtering, 4,162 of 4,515 all sequenced cells (92.2%) were utilized in the downstream analyses. **d** and **e** Violin plots showing gene count per cell grouped by each cell cluster (**d**) and genotype (**e**). **f** and **g** Violin plots showing mitochondrial ratio per cell grouped by each cell cluster (**f**) and genotype (**g**). **h** and **i** Violin plots showing read counts per cell grouped by each cell cluster (**h**) and genotype (**i**). In **d**-**i**, the data of cells passing the quality check are shown. The data are pare represented by each biological replicate in **d**, **f**, and **h**.

**
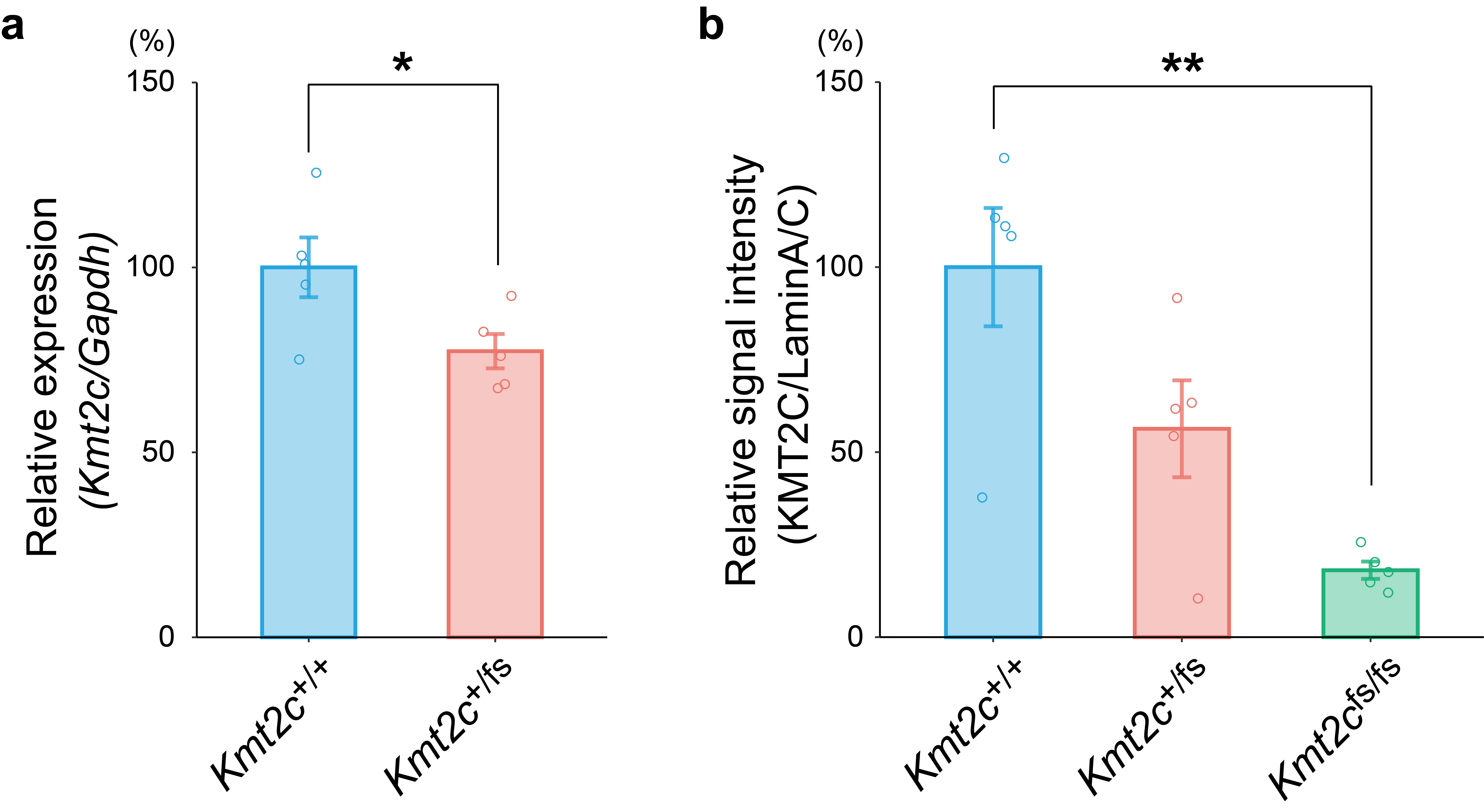
**

**Supplementary Fig. 3: Quantification of *Kmt2c* mRNA and KMT2C protein.**

**a** Quantitative analysis of *Kmt2c* mRNA by RT-qPCR. The relative expression of *Kmt2c* normalized with *Gapdh* was significantly lower in the *Kmt2c*^+/fs^ mice than in the *Kmt2c*^+/+^ mice. Total RNAs were extracted from hippocampus samples derived from adult mice (N = 5 each, 16 weeks). A Welch’s t-test was used for the statistical comparison. *P < 0.05. Data are represented as mean ± standard error mean (SEM). **b** Quantification of the signal intensity identified by the anti-KMT2C antibody. The nucleic proteins were extracted from the whole embryonic brain samples (E15.5). The intensity of the signals from the full-length KMT2C was normalized by that of the anti-LaminA/C antibody. The signal intensity of the full-length KMT2C per LaminA/C was significantly influenced by the genotypes (P = 1.55 × 10^−3^). The signal intensity of the *Kmt2c*^fs/fs^ mice was significantly lower than that of wild-type mice. One-way ANOVA with Tukey’s multiple comparisons post hoc test was utilized for the statistical comparison. P = 0.0591 for *Kmt2c*^+/+^ vs *Kmt2c*^+/fs^, P = 1.12 × 10^−3^ for *Kmt2c*^+/+^ vs *Kmt2c*^fs/fs^, and P = 0.103 for *Kmt2c*^+/fs^ vs *Kmt2c*^fs/fs^. **P < 0.01. Data are represented as mean ± standard error mean (SEM).


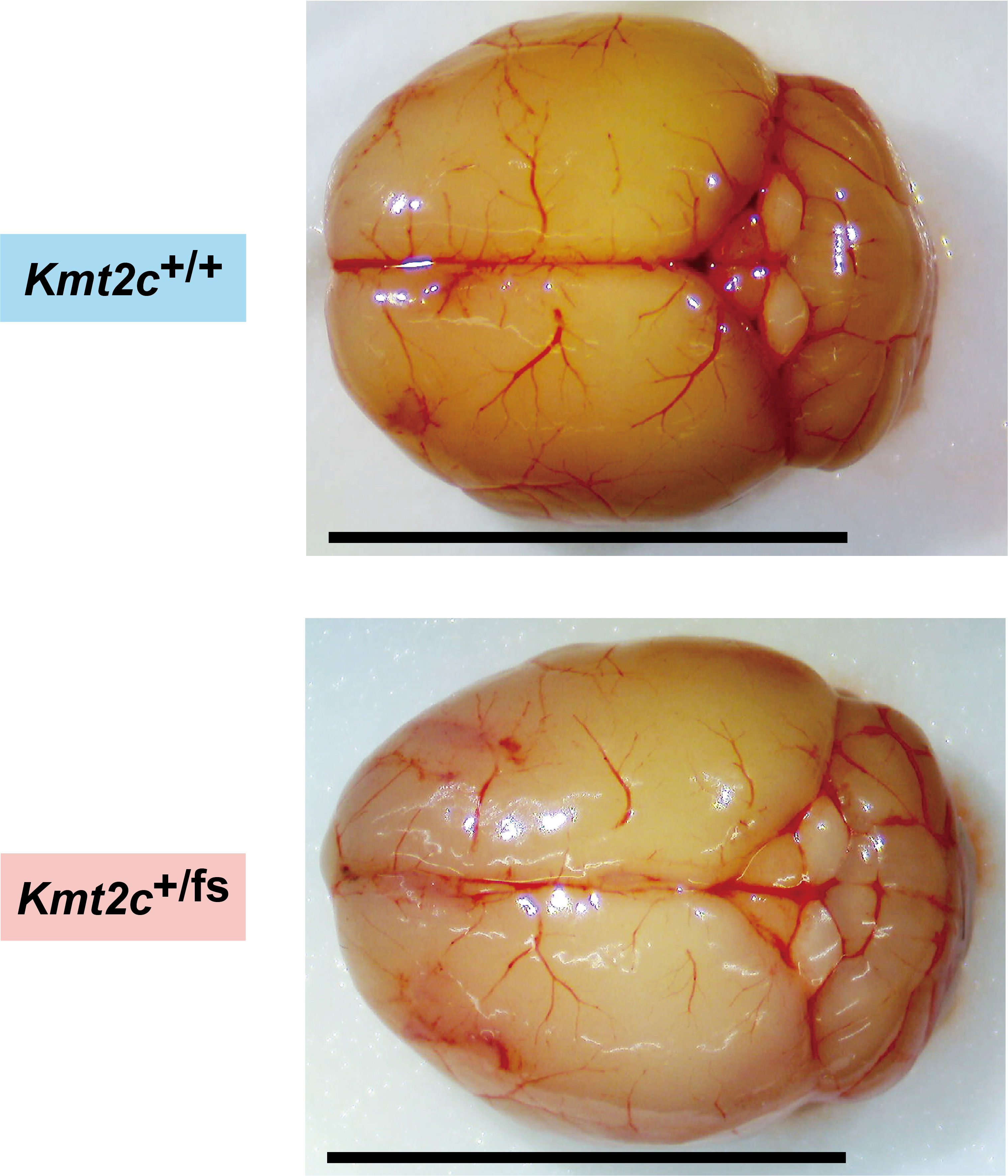


**Supplementary Fig. 4: Brains of the *Kmt2c*^+/+^ and *Kmt2c*^+/fs^ mice.**

The representative images of the fresh brains dissected from the *Kmt2c*^+/+^ and *Kmt2c*^+/fs^ mice (13 weeks old) are shown. The images were acquired by Stemi 305 stereo microscope (Carl Zeiss AG, Baden-Württemberg, Germany). Scale bar, 1 cm.

**

**

**Supplementary Fig. 5: Other results of the behavioral test battery.**

**a** Schedule of the behavioral test battery. GH - check of general health, OF - open field test, EPM - elevated plus maze, LD - light-dark transition test, RR - rotor rod test, PPI - prepulse inhibition test, HP - hot plate test, FS - Porsolt forced swim test, BM - Barnes maze test, FC - fear conditioning test, TS - tail suspension test. **b** No significant differences in general health. The *Kmt2c*^+/fs^ mice did not show abnormal body temperature (left, P = 0.222), motor performance in the wire hang test (center, *P* = 0.176), and grip strength (right). (N = 15, P = 0.130). Welch’s t-test was used for the statistical comparison. Data are represented as mean ± standard error mean (SEM). **c** The *Kmt2c*^+/fs^ mice did not show any alteration in basal activity in the open field test. There was no significance in the total distance (P = 0.364) suggesting that the *Kmt2c*^+/fs^ mice are not altered in their locomotor activity. Total center time was also not significant (*P* = 0.667). N= 30 each including mice with vehicle treatment. Welch’s t-test was used for the statistical comparison. Data are represented as mean ± standard error mean (SEM). **d** and **e** Anxiety-like behaviors were not observed. There were no significant differences in stay time in the open arm of the elevated plus maze test (P = 0.507) and in the light room of the light-dark transition test (P = 0.231). N=15 per group. Welch’s t-test was used for the statistical comparison. Data are represented as mean ± standard error mean (SEM). **f** Locomotor functions are not impaired in the *Kmt2c*^+/fs^ mice in the rotor rod test. The latency to fall in the rotor rod test was not different between the *Kmt2c*^+/+^ and *Kmt2c*^+/fs^ mice. N=15 per group. Interaction, P = 0.293; Genotype, P = 0.526; Trial, P = 1.67 × 10^−10^ A two-way repeated-measures analysis of variance (ANOVA) test was used for the statistical comparison. Data are represented as mean ± standard error mean (SEM). **g** Acoustic startle response, and prepulse inhibition. The *Kmt2c*^+/fs^ mice showed significantly lower acoustic startle response (left, *, P < 0.05/4 = 0.0125, **, P < 0.01/4 = 0.0025 (Bonferroni threshold)). It suggests insensitivity of the *Kmt2c*^+/fs^ mice against startle stimulus. The auditory sensation might be not impaired because the *Kmt2c*^+/fs^ mice properly manage the fear conditioning test with cues of tone. Though the PPI is increased in the *Kmt2c*^+/fs^ mice (right, *, P < 0.0125, **, P < 0.0025 (Bonferroni threshold)), it is not reliable results because the basal startle response is altered. N= 30 each including mice with vehicle treatment. Welch’s t-test was used for the statistical comparison comparing each decibel. Data are represented as mean ± standard error mean (SEM). dB, decibel. **h** No significance of pain response in the hot plate test. There was no significant difference in latency to a reaction between the *Kmt2c*^+/+^ and *Kmt2c*^+/fs^ mice. N=15 per group. P = 0.977. Welch’s t-test was used for the statistical comparison. Data are represented as mean ± standard error mean (SEM). **i** Depressive-like behavior was not exhibited in the *Kmt2c*^+/fs^ mice in the Porsolt’s forced swim test. The *Kmt2c*^+/fs^ mice did not show any differences in immobile time compared to that of the *Kmt2c*^+/+^ mice in both of first and second trials (first, P = 0.0853; second, P = 0.488). N=15 per group. Welch’s t-test was used for the statistical comparison. Data are represented as mean ± standard error mean (SEM). **j** Spatial memory is not impaired according to the training session of the Barnes maze test. The time to find the goal in the Barnes maze test is correctly reduced depending on the number of trials. N=15 per group. Interaction, *P* = 1.00; Genotype, P = 0.57; Trial, P = 7.87 × 10^−51^. A two-way repeated-measures ANOVA test was used for the statistical comparison. Data are represented as mean ± standard error mean (SEM). **k** No significance in the fear conditioning test. There was not any significance in the response of the *Kmt2c*^+/fs^ mice to an electronic stimulus (shock) and cues by tone stimulus in a contextual box (Conditioning). 24 hours after the conditioning, the *Kmt2c*^+/fs^ mice did not show any differences in the response when they moved to the contextual box (Context). The *Kmt2c*^+/fs^ mice showed no alterations when the mice were exposed to the cues without context 48 hours after the conditioning (Cued). Conditioning, P = 0.0574. Context, P = 0.773. Cued, P = 0.935. Welch’s t-test was used for the statistical comparison. Data are represented as mean ± standard error mean (SEM). **l** The *Kmt2c*^+/fs^ mice exhibited no depressive-like state in the tail suspension test. The percent of immobile time was not varied between the *Kmt2c*^+/+^ and *Kmt2c*^+/fs^ mice. N=15 per group. P = 0.0576. Welch’s t-test was used for the statistical comparison. Data are represented as mean ± standard error mean (SEM).

**
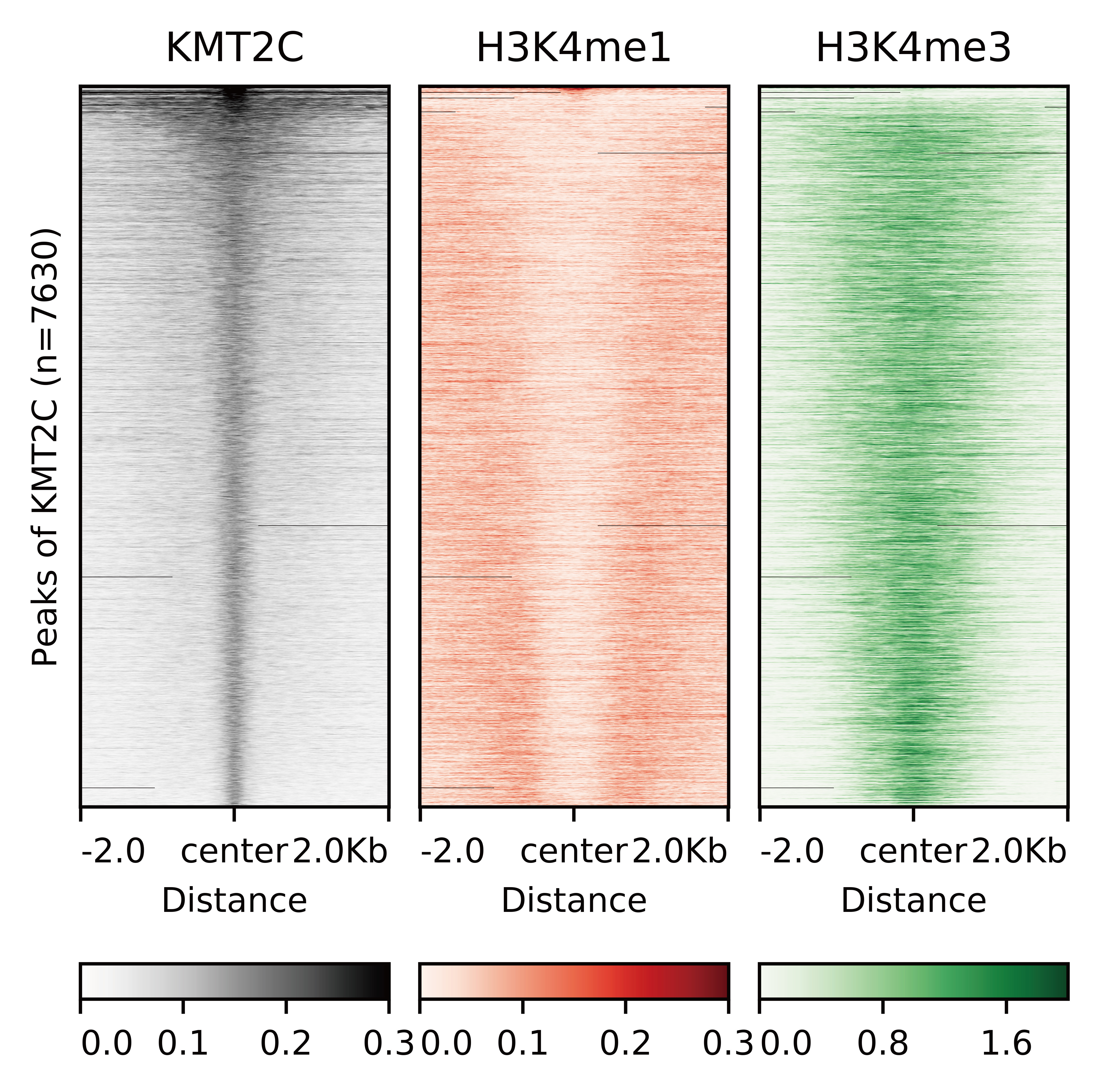
**

**Supplementary Fig. 6: Heatmap of the ChIP-seq comparing the KMT2C peaks and the data of H3K4me1/3 in ENCODE.**

The result that the signals of H3K4me3 were identified in the regions of the KMT2C peaks was reproduced if the ChIP-seq data of murine cortical samples acquired from ENCODE (https://www.encodeproject.org/) was utilized. The order of peaks was sorted depending on the signal intensity of the KMT2C peaks.

**
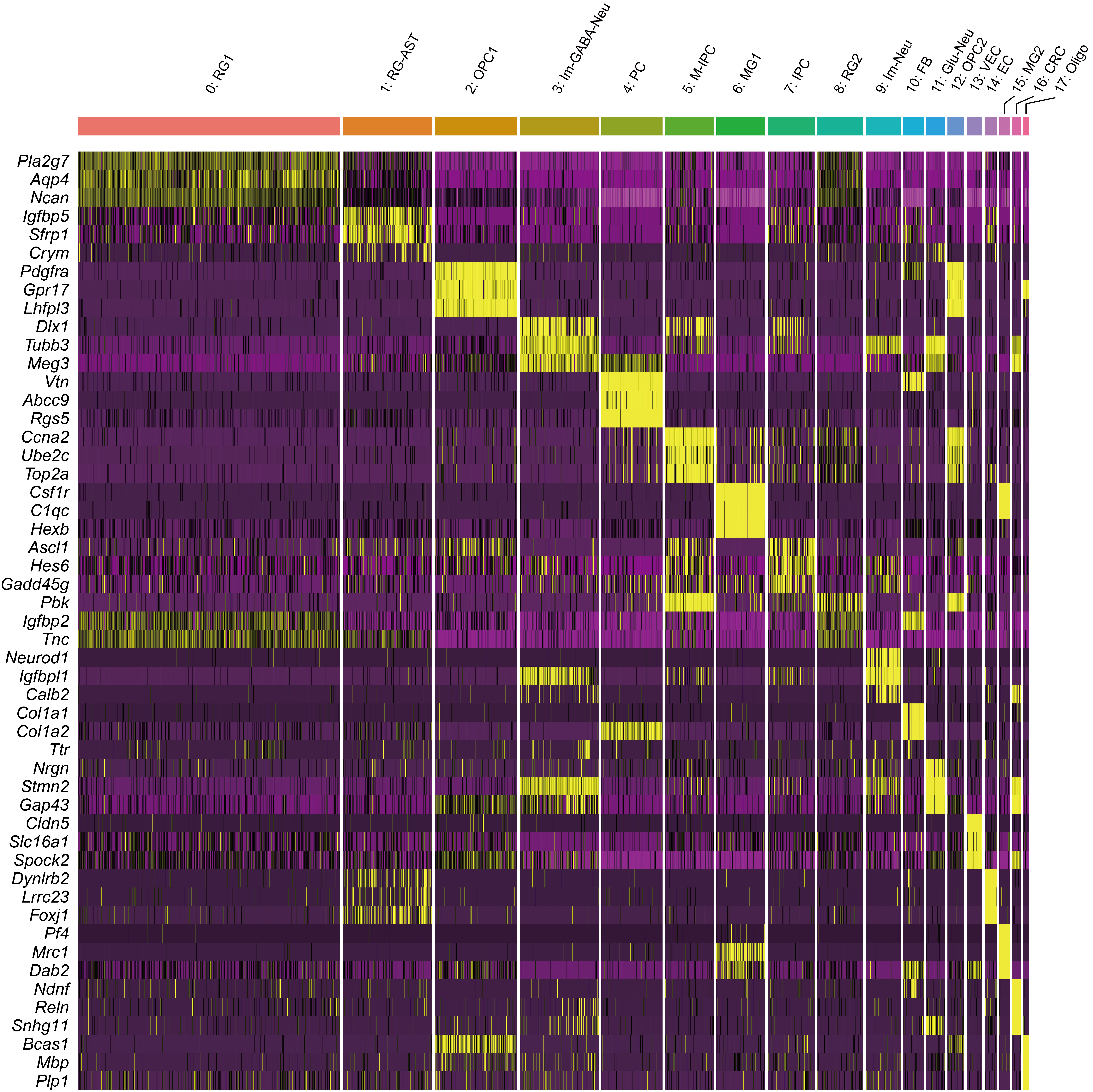
**

**Supplementary Fig. 7: Heatmap of marker genes of cell clusters identified by scRNA-seq**

The top 3 marker genes of each cell cluster were shown as representative. 0: RG1 - Radial glia subtype 1, 1: RG-AST - Radial glia ~ Astrocyte, 2: OPC1 - Oligodendrocyte precursor cell subtype 1, 3: Im-GABA-Neu - Immature GABAergic neuron, 4: PC - Pericyte, 5: M-IPC - Mitotic intermediate progenitor cell, 6: MG1 - Microglia subtype 1, 7: IPC - Intermediate progenitor cell, 8: RG2 - Radial glia subtype 2, 9: Im-Neu - Immature neuron, 10: FB - Fibroblast, 11: Glu-Neu - Glutamatergic neuron, 12: OPC2 - Oligodendrocyte precursor cell subtype 2, 13: VEC - Vascular endothelial cell, 14: EC - Ependymal cell, 15: MG2 - Microglia subtype 2, 16: CRC - Cajal-Retzius cell, 17: Oligo - Mature oligodendrocyte.

**
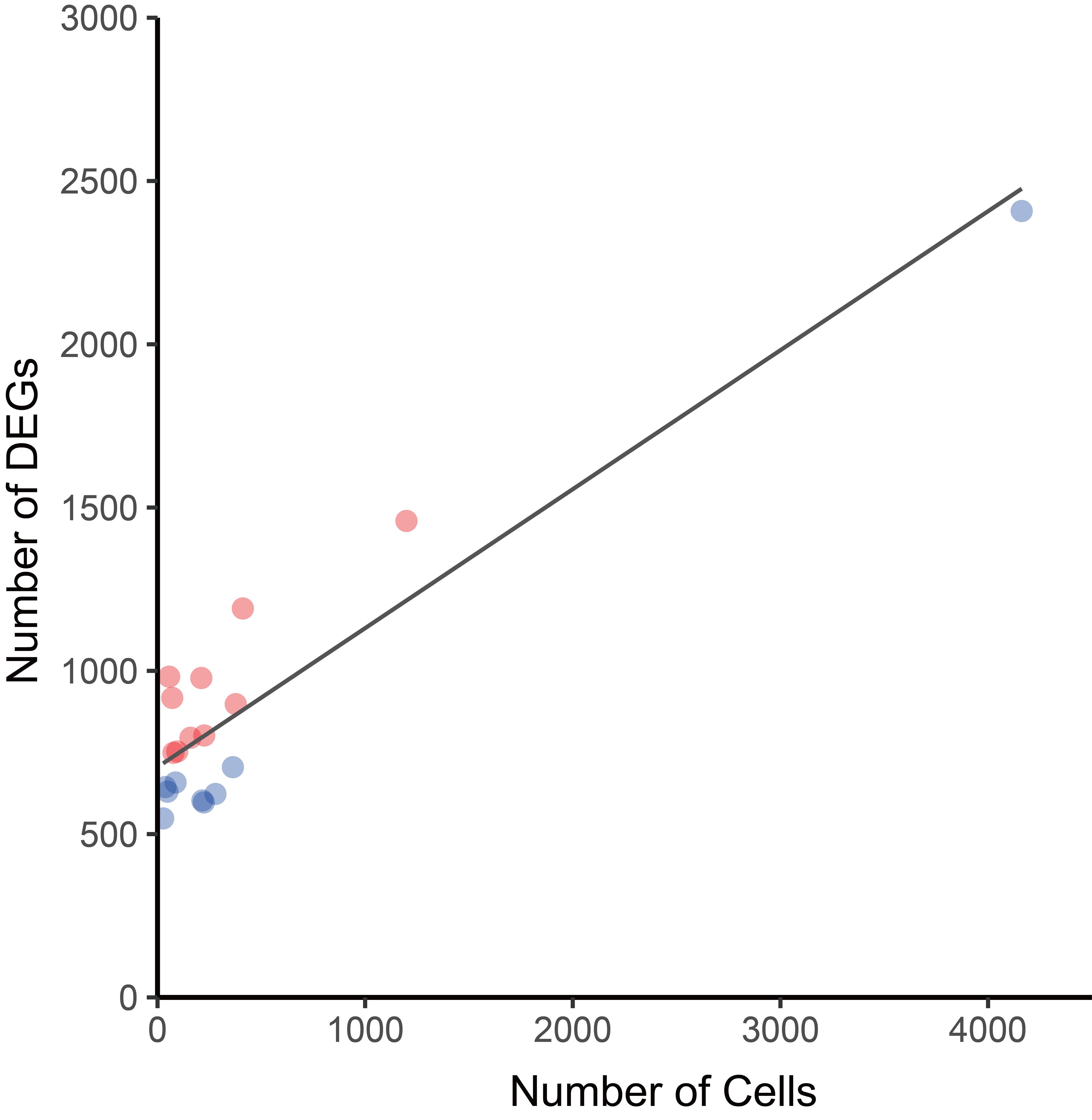
**

**Supplementary Fig. 8: Scatter plot of the number of cells and all DEGs of each cell cluster**

Scatter plot of the number of cells and all DEGs among 19 cell clusters (clusters 0−17 and pseudo-bulk). The number of DEGs with uncorrected P < 0.05 and cells were significantly correlated (Pearson’s correlation coefficient r = 0.925, *P* = 1.50 × 10^−8^). The gray line indicates the regression line. Red and blue dots represent the cell clusters that showed a larger or smaller number of observed DEGs than the expected number of DEGs calculated by the regression line, respectively.

**

**

**Supplementary Fig. 9: Gene ontology analyses of cluster 9: Im-Neu and cluster 8: RG2.**

**a** Top 5 significantly enriched GO terms in the upregulated genes of cluster 9: Im-Neu. The gray dotted line indicates the Benjamini-Hochberg (BH) corrected P value < 0.05. The black bars are the significant but excluded term in the network visualization in **c**. The complete list of relevant GO terms are shown in Supplementary Table 5i. **b** Top 5 significantly enriched GO terms in the downregulated genes of cluster 9: Im-Neu. The gray dotted line indicates the BH-corrected P value < 0.05. The color of the bars corresponds to that of the clusters shown in **c**. The complete list of relevant GO terms are shown in Supplementary Table 5j. **c** Network visualization of the significant GO terms of the upregulated genes of cluster 9: Im-Neu. GO analysis and the visualization was performed by Metascape and Cytoscape respectively, using the significant DEGs (uncorrected P value < 0.05). The size of nodes indicates the significance (-log10(BH-corrected P value)). The edge width shows similarity scores calculated by Kappa-test between the nodes, and the threshold of the score of the edges is more than 0.6. Each color of nodes indicates the same cluster. The network consisting of less than 3 nodes was excluded. The downregulated genes did not show any GO network fulfilling the above criteria. **d** Top 5 significantly enriched GO terms in the upregulated genes of cluster 8: RG2. The gray dotted line indicates the BH-corrected P value < 0.05. The black bars are the significant but excluded term in the network visualization in **f**. The complete list of relevant GO terms are shown in Supplementary Table 5k. **e** Top 5 significantly enriched GO terms in the downregulated genes of cluster 8: RG2. The gray dotted line indicates the BH-corrected P value < 0.05. The color of the bars corresponds to that of the clusters shown in **f**. The black bar is the significant but excluded term in the network visualization in **f**. The complete list of relevant GO terms is shown in Supplementary Table 5l. **f** Network visualization of the significant GO terms of the upregulated genes. The criteria of the visualization were the same with **c**. The downregulated genes did not show any GO network fulfilling the above criteria.

**
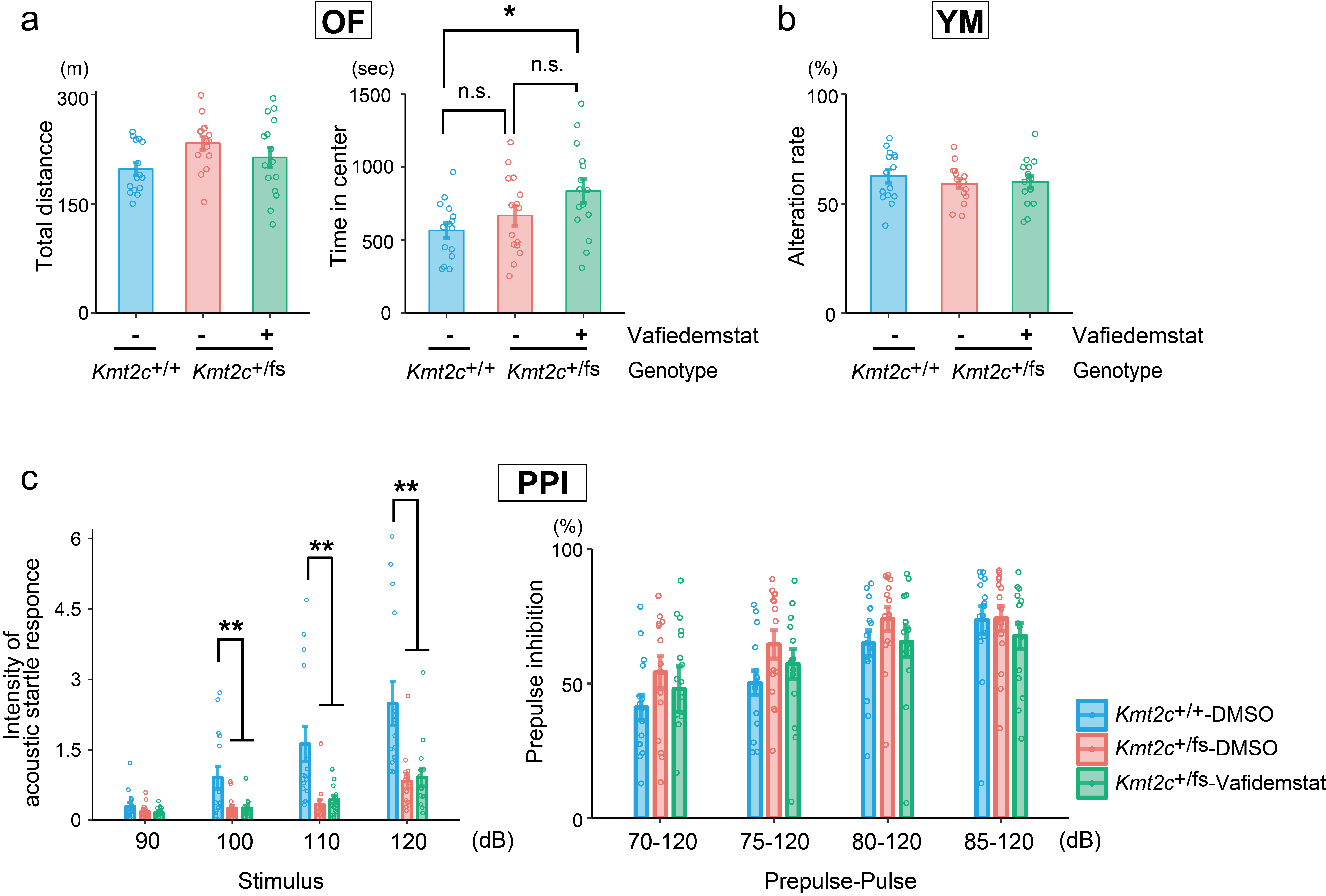
**

**Supplementary Fig. 10: Behavioral tests with the treatment of vafidemstat.**

**a** The significance in the open field (OF) test was not reproduced in the pharmacological test on a smaller scale. The significance of stay time in the central region was not detected without the data of the behavioral test battery in Fig. 1. The rescue effect could not be evaluated. (Total distance, P = 0.0789; Time in the center, P = 0.0276). A one-way ANOVA test was used for the statistical comparison. The post hoc test was performed by Tukey’s test. Data are represented as mean ± standard error mean (SEM). **b** The alteration rate in Y-maze (YM) test was not lower in the *Kmt2c*^+/fs^ mice in the test of vafidemstat with small-scale samples. The significance of the Y-maze test was not found in the test with vafidemstat. P = 0.642. A one-way ANOVA test was used for the statistical comparison comparing each decibel. Data are represented as mean ± standard error mean (SEM). dB, decibel. **c** Acoustic startle response in prepulse inhibition (PPI) was not rescued by vafidemstat. The acoustic startle response was significantly lower in the *Kmt2c*^+/fs^ mice without the data of conventional behavioral tests in Fig.1 although the rescue effects were not found. The p-values of ASR are shown below. 90db, P = 0.129; 100db, P = 3.38 × 10^−10^; 110db, P = 3.41 × 10^−10^; 120db, P = 4.65 × 10^−10^. PPI did not show any significance (70db, P = 0.384; 75db, P = 0.161; 80db, P = 0.350; 120db, P = 0.591). A one-way ANOVA test was used for the statistical comparison comparing in each decibel. *, P < 0.05, *, P < 0.01. The post hoc test was Tukey’s test. Data are represented as mean ± standard error mean (SEM). dB, decibel.

**Supplementary Table List**

Supplementary Table 1: Summary of PCR conditions in this study

Supplementary Table 2: Summary data regarding the bulk RNA-seq of the forebrain

Supplementary Table 3: Lists of genes differentially expressed in each cluster in the scRNA-seq

Supplementary Table 4: Peaks of KMT2C and DEGs in the microarray analysis

Supplementary Table 5: Summary data regarding the prioritization of the cell clusters in the scRNA-seq

Supplementary Table 6: Peaks of SETD1A in promoter-TSS regions

Supplementary Table 7: Summary data regarding the pharmacological analysis with vafidemstat
