# Supplementary material for: Transcriptomic dysregulation and autistic-like behaviors in Kmt2c haploinsufficient mice rescued by an LSD1 inhibitor
Source: Mol Psychiatry. 2024 Mar 26;29(9):2888–904. doi: 10.1038/s41380-024-02479-8 (PMC11420081; doi:10.1038/s41380-024-02479-8)
Supplement: Supplementary file 2 — Supplementary Method [file 41380_2024_2479_MOESM2_ESM.docx]

**Supplementary Method**

**Transcriptomic dysregulation and autistic-like behaviors in *Kmt2c* haploinsufficient mice rescued by an LSD1 inhibitor**

Takumi Nakamura^1, 2, 3, 4^, Toru Yoshihara^5^, Chiharu Tanegashima^6^, Mitsutaka Kadota^6^, Yuki Kobayashi^7^, Kurara Honda^1^, Mizuho Ishiwata^2, 3^, Junko Ueda^1, 3^, Tomonori Hara^1, 8^, Moe Nakanishi^9, 10^, Toru Takumi^9, 11^, Shigeyoshi Itohara^7^, Shigehiro Kuraku^6, 12^, Masahide Asano^5^, Takaoki Kasahara^3, 13^, Kazuo Nakajima^2, 3, 14^, Takashi Tsuboi^4^, Atsushi Takata^1, 15, *^ & Tadafumi Kato^2, 3 *^

*1: Laboratory for Molecular Pathology of Psychiatric Disorders, RIKEN Center for Brain Science, Saitama, Japan.*

*2: Department of Psychiatry and Behavioral Science, Juntendo University Graduate School of Medicine, Tokyo, Japan.*

*3: Laboratory for Molecular Dynamics of Mental Disorders, RIKEN Center for Brain Science, Saitama, Japan.*

*4: Department of Life Sciences, Graduate School of Arts and Sciences, The University of Tokyo, Tokyo, Japan*

*5: Institute of Laboratory Animals, Kyoto University Graduate School of Medicine, Kyoto, Japan*

*6: Laboratory for Phyloinformatics, RIKEN Center for Biosystems Dynamics Research, Hyogo, Japan*

*7: Laboratory for Behavioral Genetics, RIKEN Center for Brain Science, Saitama, Japan*

*8: Department of Organ Anatomy, Tohoku University Graduate School of Medicine, Miyagi, Japan*

*9: Laboratory for Mental Biology, RIKEN Center for Brain Science, Saitama, Japan*

*10: Laboratory for Molecular Mechanism of Brain Development, RIKEN Center for Brain Science, Saitama, Japan*

*11: Department of Physiology and Cell Biology, Kobe University School of Medicine, Hyogo, Japan*

*12: Molecular Life History Laboratory, Department of Genomics and Evolutionary Biology, National Institute of Genetics, Shizuoka, Japan*

*13: Institute of Biology and Environmental Sciences, Carl von Ossietzky University of Oldenburg, Oldenburg, Germany*

*14: Department of Physiology, Teikyo University School of Medicine, Tokyo, Japan*

*15: Research Institute for Diseases of Old Age, Juntendo University Graduate School of Medicine, Tokyo, Japan*

***Correspondence should be addressed to:**

Atsushi Takata, M.D., Ph.D.

Laboratory for Molecular Pathology of Psychiatric Disorders, RIKEN Center for Brain Science, 2-1 Hirosawa, Wako, Saitama, 351-0198, Japan

Phone & Fax: +81-48-467-9703

Email: atsushi.takata@riken.jp

Tadafumi Kato, M.D., Ph.D.

Department of Psychiatry and Behavioral Science, Juntendo University Graduate School of Medicine, 2-1-1 Hongo, Bunkyo-ku, Tokyo, 113-8421, Japan

Phone & Fax: +81-3-5802-1071

Email: [tadafumi.kato@juntendo.ac.jp](mailto:tadafumi.kato@juntendo.ac.jp)

**Behavioral test battery**

The behavioral tests were performed using the several behavioral test apparatuses described below. All apparatuses without a manufacturer's name in the following text were manufactured by O'HARA & CO., LTD., Tokyo, Japan. Other detailed information such as sizes of the experimental apparatus, illumination conditions, etc. is reported by Sukegawa et al., (2022). DOI: 10.1111/ejn.15602.

**General health and neurological examination**

General health and neurological examinations were performed prior to the test battery. The tests on the first day included rectal temperature, whisker state, coat state, righting reflex, whisker twitch, ear twitch, and reaching were diagnosed manually. The measurement of rectal temperature and wire hang test was conducted on the same day. On the next day, grip strength and the symptoms of epilepsy were examined.

**Open-field test**

Spontaneous activities of the mice were measured in the open-field chamber (W 407 mm, D 407 mm, and H 305 mm; Accuscan Instruments, Ohio, USA) The animals could freely explore the field for 60 min. The total distance traveled and time spent in the center area (inner 30% area) were recorded.

**Y-maze**

The Y-maze test was performed in an apparatus with three arms arranged at 120º intervals for 5 min. The total distance was not impaired in the mice in this paper. The alternation rate (the number to enter all three arms within three entries/ [the total number of entries into arms] -2) was recorded.

**Light-dark transition test**

The light-dark transition test was conducted in the light-dark-separated area box for 10 min. The chamber was equally divided into two chambers by a wall having a hole that allowed animals to move freely between light and dark areas. Animals were positioned in the dark chamber at the start of the experiment. The latency to stay in the light chamber was recorded.

**Elevated-plus maze**

A test chamber for the elevated plus maze test has four arms arranged at 90º connected to the center area. Two of the arms were surrounded by transparent walls (closed arm) and others were not surrounded (open arm). Animals were positioned in the center area when the experiment was started and allowed to move freely for 10 min. The time spent in each arm was recorded.

**Rotarod test**

In the rotarod test, six trials (3 trials/day 2 days) were conducted using a rotarod apparatus (Ugo Basile, Varese, Italy). Animals were positioned on a rotating rod (4 rpm, 30 mm in diameter) at the start of the experiment. The speed of rotation was gradually accelerated from 4 to 40 rpm over 5 min. The latency to fall was recorded.
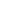


**Hot plate test**

Animals were positioned on a hot plate apparatus (Columbus Instruments International., Ohio, USA) at 55ºC in the hot plate test. Animals moved freely on the hot plate during the test. The latency to the first foot shake or paw lick was recorded.

**Crawley’s three-chamber social interaction test**

The social behavior of animals was measured in Crawley’s social interaction test chamber. The chamber consisted of three areas separated by transparent walls with holes, and they could freely move three areas. On the first day, all stranger mice were habituated to the small cages in the arena for 10 min. On the second day, subject animals were first placed in the center area with a stranger caged mouse on one side, for 10min (mouse cage vs. empty cage). The duration of the area staying was recorded.

**Porsolt’s forced swim test**

A small round pool was utilized for the Porsolt’s forced swim test. The pool was filled with 20ºC hypochlorous acid water to a height of 75 mm not to touch the animal’s rear foot. The mice were analyzed for two consecutive days. Animals were allowed to swim freely in the pool for 10 min. The percent of the immobile time, that is, floating status, was recorded.

**Prepulse inhibition test**

The prepulse inhibition was measured in a startle reflex measurement box. Acoustic startle responses were quantified by stimuli of 90, 100, 110, and 120 dB of white noise (40 ms, 1000–20,000 Hz). Subsequently, prepulse inhibition of acoustic startle responses was measured by pairs of 70 (pre)–120 dB, 75–120 dB, 80–120 dB, and 85–120 dB of white noise (40 ms). The inhibition rate was calculated in each prepulse condition.

**Barnes maze**

The Barnes maze was performed on a white circular arena. Twelve holes (40 mm in diameter) were equally spaced around its circumference and one escape box was set under the specific hole for each mouse. The training session consisted of 20 trials (one or two trials/day, 5 min). After 1 day and 8 days of the training sessions, probe tests were conducted.

**Fear-conditioning test**

The fear-conditioning test took 3 days. On the first day, conditioning was conducted in box A. The electrical foot shock and cued tone were simultaneously presented three times during this conditioning phase. On the second day, a contextual test was conducted in box A without shock and cued tone. On the third day, a cue test was conducted in the different box B and the cued tome was presented at certain times in the trial. The duration of immobile time (freezing duration) was recorded in each trial.

**Tail suspension test**

The base of the tail of the mouse was taped onto a metal board, and animals were suspended 270 mm above the floor in the tail suspension test. The percent of the immobile time in 10 min was recorded.

**IntelliCage analyses**

The procedures of the IntelliCage (TSE Systems, Inc. Chesterfield, MO, USA) analysis referenced our previous report. IntelliCage apparatus (39 × 58 × 21 cm) has four chambers in each corner accessible through an open doorway. The doorway has a ring antenna and LED lights. Two doors in each chamber were used to control access to water bottles. A radiofrequency identification transponder (Standard Microchip T-VA, DataMars, Lamone, Switzerland; and Trovan, Melton, UK) was implanted into the dorso-cervical region of mice under isoflurane inhalation anesthesia in order to track each mouse in the corner chambers. The condition of light was as follows: the light period, 08:00–20:00 local time, and the dark period, 20:00–08:00 (Light–Dark 12:12 h).

The order of the test battery is the following: free adaptation (2 days), Nose poke adaptation (3 days), drinking session adaptation (3 days), place learning test (7 days), place learning reversal test (5 days), impulsivity test (4 days), attention test (5 days), place avoidance test (9 days), delay discounting test (10 days) and serial reversal test (24 days). Seven wild-type and seven mutant mice of the same sex of each mouse line were kept and analyzed in each IntelliCage. Testing began at 16 weeks of age. The details of each phase and test are as follows.

**Free adaptation**

To adapt the mice to the IntelliCage, all water bottles were always and freely accessible to allow mice.

**Nose poke adaptation**

Mice could drink water by opening the gate that closed the bottles with a nose poke. The doors were open for 5 s after a nose poke. The gates could be always opened.

**Drinking session adaptation**

The gates were opened for 5 s in the drinking session phase (21:00–24:00 local time) after a nose poke.

**Place learning test**

Each mouse could drink in only one of the four corners (correct corner) at 21:00–24:00 local time. The visit rates with nose poke to the correct corner were calculated every day.

**Place learning reversal test**

The correct corner was moved to the diagonally opposite corner of the correct corner in the place learning test. The correct rates were quantified in the same way as in the place learning test.

**Impulsivity test**

In the training step, doors were opened for 5 s if a nose poke was performed 1, 2, or 4 s after they enter to the chambers. In the test step, the doors were not opened if the mice performed a nose poke within 2 s after entering the chambers (premature nose poke). If a nose poke was performed for more than 2 s after entering, the doors were opened. This test was accessible from 21:00 to 04:00 local time. Omissions were defined as ‘Neutral’ nose pokes with a visit duration of fewer than 2 s, and premature nose pokes were defined as ‘Incorrect’ nose pokes. To calculate each rate, the number of each category was divided by the total number of nose pokes. The total number was the sum of the number of correct nose pokes, premature nose pokes, and omissions.

**Attention test**

During the training step, one of the LED lights above the gates (correct) was turned on for 0.5, 1, or 2 s randomly. After the nose poked to the ‘correct’ gate, the gate was opened and the bottles were accessible. In the test step, one of the two LED lights was turned on at random for 0.3, 0.5, or 1, randomly, 4 s after entering the chambers. The correct rate was defined by the number of ‘correct’ nose pokes per the total number of nose pokes. For statistical analyses, the average of the correct rate over 5 days was utilized.

**Place avoidance test**

Mice were exposed to an air puff if they visited one of the four corners (air puff corner) on the first day. The mice were transferred to their home cages 24 h after the training. Another 24 h after the transfer, all mice were returned to the IntelliCages for the test. The visit rates to air puff corners per visit the corner were calculated daily.

**Delay discounting test**

In this test, the water in one of the two bottles in each corner was exchanged for 0.5% saccharin water, which is preferred by mice. The opening of the door to the bottle with saccharin water was delayed for 0-8 s. In the training step, the delay time of saccharin water was 0 s to adapt the mice to prefer saccharin water. Delays to open the doors were increased by 1 s every 24 h in the test step. The rate of drinking saccharin water was calculated every day. The bottles were accessible anytime.

**Serial reversal test**

The procedure of the serial reversal test is described below. As a training step, one of the four corners (correct corner) is accessible to drink water (place learning test). 7 days after the place learning test, the correct corner was moved to the diagonally opposite corner to investigate the reversal learning and train mice to learn the moving of the correct corner (place learning reversal test). The serial reversal test was started 5 days after the place learning reversal test. The correct corner was moved to the other diagonal line on the first day of the serial reversal test. Afterward, the correct corner was changed to the corner diagonally opposite to the corner of the previous day, every day, and this diagonal moving was continued for four days. These processes were repeated three times, and the rate of the visits to the correct corner in the previous day for the first 15 minutes was calculated as the indicator for inflexibility.
